# BIIDXI, a DUF642 Cell Wall Protein That Regulates Pectin Methyl Esterase Activity, Is Involved in Thermotolerance Processes in *Arabidopsis thaliana*

**DOI:** 10.3390/plants11223049

**Published:** 2022-11-11

**Authors:** Eduardo Pineda-Hernández, José Erik Cruz-Valderrama, Ximena Gómez-Maqueo, Eleazar Martínez-Barajas, Alicia Gamboa-deBuen

**Affiliations:** 1 Instituto de Ecología, Universidad Nacional Autónoma de México (UNAM), Ciudad de México 04510, Mexico; 2 Facultad de Química, Universidad Nacional Autónoma de México (UNAM), Ciudad de México 04510, Mexico

**Keywords:** plant cell wall, thermotolerance, DUF642 proteins, pectin methyl esterases

## Abstract

Plant cell wall remodeling is an important process during plant responses to heat stress. Pectins, a group of cell wall polysaccharides with a great diversity of complex chemical structures, are also involved in heat stress responses. Enzymatic activity of the pectin methyl esterases, which remove methyl groups from pectins in the cell wall, is regulated by DUF642 proteins, as described in different plants, including *Arabidopsis thaliana* and *Oryza sativa*. Our results demonstrated that heat stress altered the expression of the DUF642 gene, *BIIDXI*. There was an important decrease in *BIIDXI* expression during the first hour of HS, followed by an increase at 24 h. *bdx*-1 seedlings had less tolerance to heat stress but presented a normal heat stress response; *HSFA2* and *HSP22* expressions were highly increased, as they were in WT seedlings. Thermopriming triggered changes in pectin methyl esterase activity in WT seedlings, while no increases in PME activity were detected in *bdx*-1 seedlings at the same conditions. Taken together, our results suggest that BIIDXI is involved in thermotolerance via PME activation.

## 1. Introduction

The cell wall has a complex structure, formed mainly by the polysaccharide cellulose, hemicelluloses and pectins, and by proteins from different families. The cell wall actively participates during the different stages of plant development and in response to abiotic and biotic stresses [1]. High temperatures induce heat stress by promoting important alterations in plant growth and development [2]. Plants exposed to sublethal temperatures acquire thermotolerance, a mechanism that allows survival at higher temperatures that are normally lethal. Under laboratory conditions, thermotolerance acquisition can be induced using thermopriming treatment. In addition to the accumulation of heat shock proteins (HSPs), plant cell wall remodeling has also been considered as an integral part of the heat response network, as it was recently demonstrated for salt-induced responses [3,4]. In the mold *Aspergillus fumigatus*, cell wall stress generated by heat stress actively participates in the perception and signaling of stress, triggering the coordinated expression of heat shock transcription factors (HSFs) and HSPs [5].

Heat stress may also promote mechanisms to resist recurrent heat stress events, i.e., thermomemory. The participation of HSFs, HSPs, primary metabolism enzymes, growth regulators, and epigenetic processes in thermomemory has been widely described [2]. The involvement of plant cell wall remodeling processes in thermomemory has not yet been described but is suggested by the upregulation of plant cell wall protein-encoding genes by the transcription factor HSFA2 [6].

The plant cell wall polysaccharides known as pectins are the most sensitive to the mechanical deformation that occurs in plant cells during the different stages of development and during the responses to biotic and abiotic stresses [3,7]. Pectins have a great diversity of complex chemical structures. Among the five different groups of pectins, homogalacturonans (HGs) are the most abundant. These polysaccharides are linear chains of α-1,4-linked D-galacturonic acid (DGalA) residues whose chemical properties, determined by methyl esterification and acetyl esterification, may be influenced by development and environmental conditions [8].

Highly methyl-esterified HGs are secreted into the cell wall, where their degree of esterification is determined by the activity of pectin methyl esterases (PMEs). The removal of the methyl group by PMEs determines the degree of methylation of pectins, promoting both relaxation and increased rigidity of the cell wall: the de-methyl-esterified HGs chains can be the substrate for enzymes such as polygalacturonases (PGs) and pectate lyases (PLs) that promote cell wall relaxation; likewise, the acid groups present in the de-methyl-esterified HG chains can interact with calcium ions found in the cell wall, forming structures called “egg boxes” that confer greater rigidity to the wall [9]. The regulation of the degree of pectin-methyl esterification present in the cell wall is determined by the combined activity of PMEs and the proteins that regulate their activity: the pectin methyl esterase inhibitors (PMEIs) and the DUF642 family proteins [10,11].

PMEs constitute a multigene family in plants that are involved in different physiological processes during plant development. In *Arabidopsis thaliana*, there are 66 PME-related genes with differential expressions during plant development [12]. In *A. thaliana* seedlings, decreases in gene expression of some PMEs resulted from exposure to heat shock, and the PME7, PME28, PME34, and PME53 were observed to participate in the thermotolerance process. The seedlings of the *pme7*, *pme28*, and *pme34* mutant lines have a higher sensitivity to HS [13], while the seedlings of the *pme53* mutant line have a higher thermotolerance [14]. The increase in temperature promotes an increase in PME activity that participates in cell wall remodeling in response to heat stress, a crucial process for the acquisition of thermotolerance [3,13,15]. The increase in PME activity could be a result of their regulation through other interacting proteins, as has been suggested by Bosch and Hepler [16]; PME enzymatic activity regulation is likely more crucial than the regulation at the translation or protein accumulation level.

PMEI proteins were first identified in kiwi [17], and ever since, they have been reported to modulate PME activity in a wide variety of biological contexts, participating in cell adhesion, cell wall stress signaling, and defense, among other processes [18]. The participation of these proteins has been described in response to cold and salinity stress [19], but they are poorly studied during heat stress response, despite previous suggestions by Wu et al. [3] that specific PMEIs might be involved in the regulation of PME34 activity during this process.

The DUF642 family is a family of cell wall proteins specific to spermatophyte plants. In Arabidopsis, 10 genes have been described for this family that are grouped into four different clades (A-D) and are differentially expressed during plant development [11,20]. DUF642 proteins have been described in cell wall proteomes of different tissues in Arabidopsis and other plants [21]. The DUF642 proteins encoded by the genes *At4g32460* (*BIIDXI*) and *At5g11420* interact *in vitro* with the AtPME3 (pectin methyl esterase 3, *At3g14310*) protein [22]. The DUF642–PME protein interaction has been verified for the rice DUF642 protein, named DFOT1, and the homologue of PME3 [23]. Different tissues in the *BIIDXI* and *At2g41800* (*TEEBE*) loss-of-function and overexpression lines show alterations in PME activity. In seedlings, the *bdx*-1 and *teb*-1 mutants show a decrease in PME activity, while *OEBDX* and *GCTEB* overexpression lines show an increase [24]. For *bdx*-1 and *teb*-1, a decrease in the detection of demethyl-esterified pectins in the seed endosperm and in the epidermal cells of the hypocotyl, respectively, has also been described [25,26]. In rice, the *dfot 1* mutant showed a lower degree of methyl esterification in the cell wall of the lodicule cells, a structure involved in the opening of the flower [23].

The increase in PME activity in response to high temperature determines a remodeling of the cell wall that could participate in the perception and signaling of heat stress [3,27]. To address the role of the DUF642 proteins in the processes of thermotolerance and thermomemory through the regulation of PME activity, we first investigated the effect of different temperatures on the expression levels of three DUF642 genes: *At3g08030*, *BIIDXI*, and *DGR2 (DUF642 L-GALL RESPONSIVE GENE 2* (*At5g25460*)). We found that *bdx*-1 seedlings are more sensitive to heat, and the dynamics of total PME activity during treatment is different from that of WT plants. Our results provide evidence that the DUF642 family proteins could be involved in the regulation of PME activity under heat stress. 

## 2. Results 

### 2.1. BIIDXI Expression Decreases during Thermopriming

**Figure 1:**
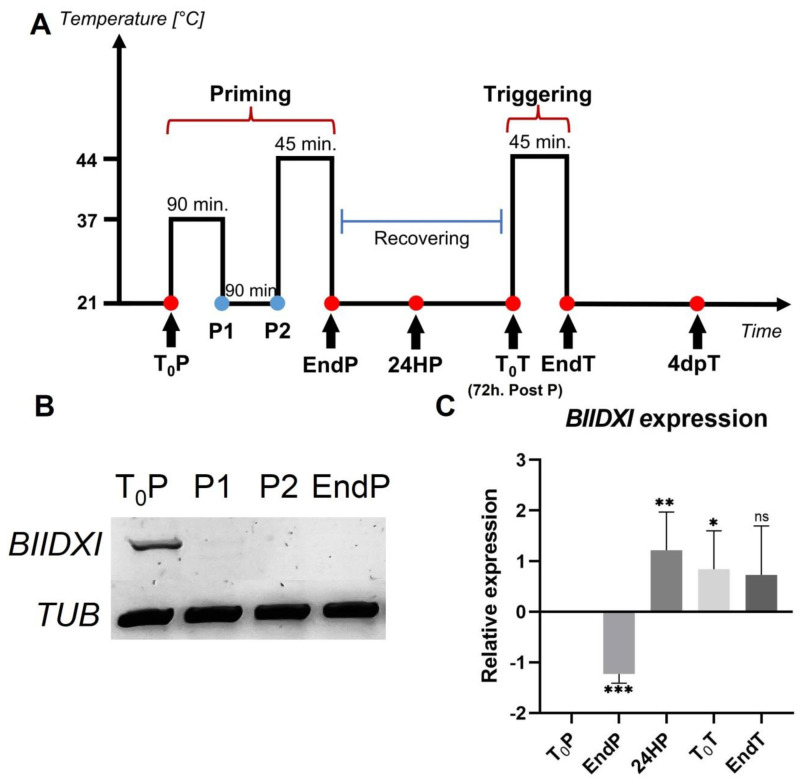
*BIIDXI* expression during thermotolerance and thermomemory. (**A**) Schematic representation of the protocol (based on Olas et al. [29]). Seedlings were collected and frozen immediately at the beginning and at the end of priming (T_0_P and EndP), 24 h after the end of priming (24HP), 72 h after priming/start of triggering (T_0_T), at the end of triggering (EndT), and 4 days after triggering (4dpT). (**B**) *BIIDXI* expression within the priming, including sublethal heat treatment (**C**) on the *BIIDXI* expression level during triggering. The expression values are relative to T_0_P expression. Error bars indicate S.D. (n = 4). Statistical analyses were performed using Student’s *t*-test. Asterisks indicate statistically significant differences to T_0_P (* *p* < 0.05, ** *p* < 0.01, *** *p* < 0.001, ns, not significant).

Heat stress decreased the expression levels of DUF642 genes from peach fruits, and a decrease in the accumulation of DUF642 proteins in peach fruits and *Brachipodium* leaves in response to heat was also reported [28]. To investigate if DUF642 genes from *A. thaliana* could be involved in thermotolerance acquisition and/or in thermomemory (Figure 1A), we first determined the expression levels of *At3g08030*, *BIIDXI*, and *At5g25460*, the DUF642 genes that are highly expressed in seedlings (Figure S1 and Figure 1B). No differences in expression levels of *At3g08030* and *At5g25460* were recorded during thermotolerance and thermomemory, described in Figure 1A. The *BIIDXI* gene expression level decreased in response to heat stress at 37 °C, with no further increase throughout the thermopriming phase (Figure 1B). At 24 h after priming, an increase in *BIIDXI* expression was observed (Figure 1C).

### 2.2. The bdx-1 Mutant Is Affected by Thermopriming Treatment

**Figure 2:**
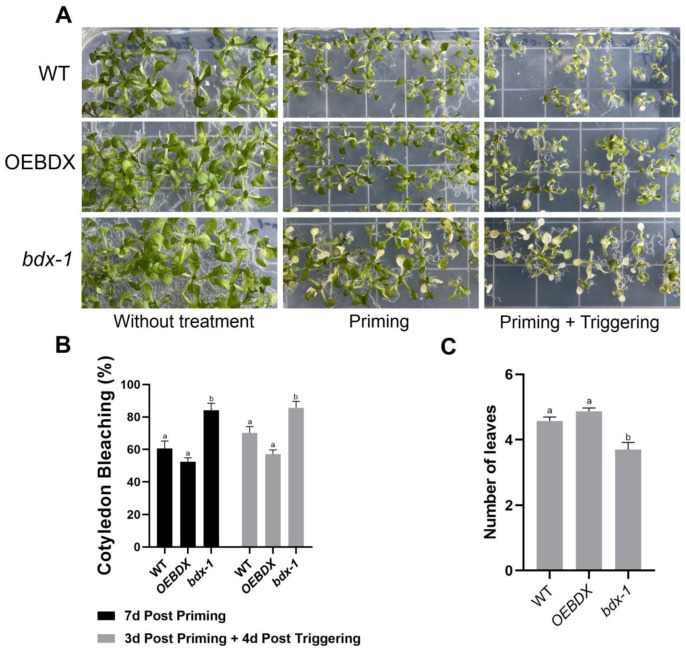
Phenotypic changes of WT, *bdx*-1, and *OEBDX* seedlings during thermotolerance and thermomemory. (**A**) WT Col-0, *bdx*-1, and *OEBDX* seedlings were grown on MS medium. Seedlings with two leaves were subjected to priming treatment (T_0_P). Seedling phenotypes of control, 7d post priming and 3d post priming, and 4d post triggering are shown. (**B**) The bleaching cotyledon phenotype was quantified in seedlings shown in (**A**) and represented as a percentage. (**C**) Leaves observed at 4d post triggering. Error bars indicate SEM (n = 3). An amount of 10–12 seedlings constituted each biological replicate. For cotyledon bleaching, statistical analyses were performed using arcsine conversion of percentages. Different letters indicate significant differences calculated using one-way ANOVA with *post-hoc* Tukey’s test (*p* < 0.05).

The differential effects of priming and triggering on *BIIDXI* gene expression suggest that this gene could be involved in thermotolerance and/or thermomemory processes, so the effect of both treatments on the development of WT, overexpression (*OEBDX*) transgenic lines, and loss-of-function transgenic line seedlings (*bdx*-1) were performed. The detrimental effects of priming and triggering on seedling development were quantified as the percentage of cotyledon bleaching in seedlings (Figure 2). After three days of priming treatment, 80% of the *bdx*-1 seedlings showed cotyledon bleaching, a significantly higher percentage than WT and *OEBDX* seedlings, which did not show significant differences between them (Figure 2A,B). Leaf number in *bdx*-1 seedlings was also significantly lower after priming, in contrast to untreated seedlings (7d post priming). Triggering did not affect the percentage of cotyledon bleaching in any of the three lines, but a lower growth of rosette leaves was observed. 

**Figure 3:**
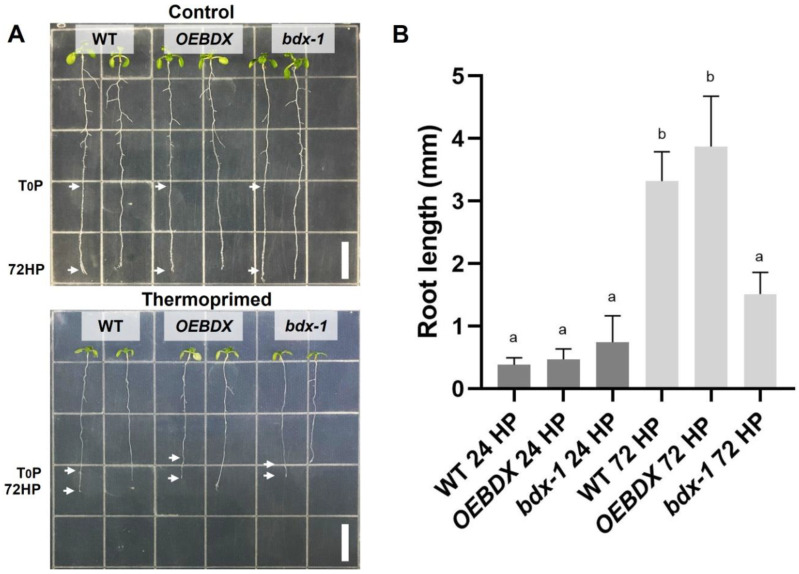
Effect of thermopriming treatment on root growth. (**A**) Seedlings of WT, *OEBDX*, and *bdx*-1 were sown vertically on MS medium. The seedlings were exposed to priming treatment, and the comparisons of the root lengths between the lines were performed 72 h after priming. (**B**) Root length average after 24 and 72 h of priming. Error bars indicate SEM (n = 3). An amount of 30 seedlings constituted each biological replicate. Different letters indicate significant differences calculated using one-way ANOVA with *post-hoc* Tukey’s test (*p* < 0.05). White bars = 10 mm.

To further investigate the effect of priming on seedling development and because of previous reports indicating that root growth is reduced in seedlings grown at 30 °C for 3–4 days [30], we determined the root length of thermoprimed seedlings. We observed that heat reduced root growth in WT, *OEBDX*, and *bdx*-1, but the length of the *bdx*-1 roots after the thermopriming was significantly reduced, with respect to the treated WT and *OEBDX* lines. At 24 h after priming (24HP), no difference in root length between lines was observed; at 72 h after priming (72HP), there was a significant increase in root length that was not observed in *bdx*-1 roots (Figure 3). These results suggest that *BIIDXI* is involved in thermotolerance acquisition.

### 2.3. BIIDXI Participates in the Regulation of PME Activity in Thermotolerance

**Figure 4:**
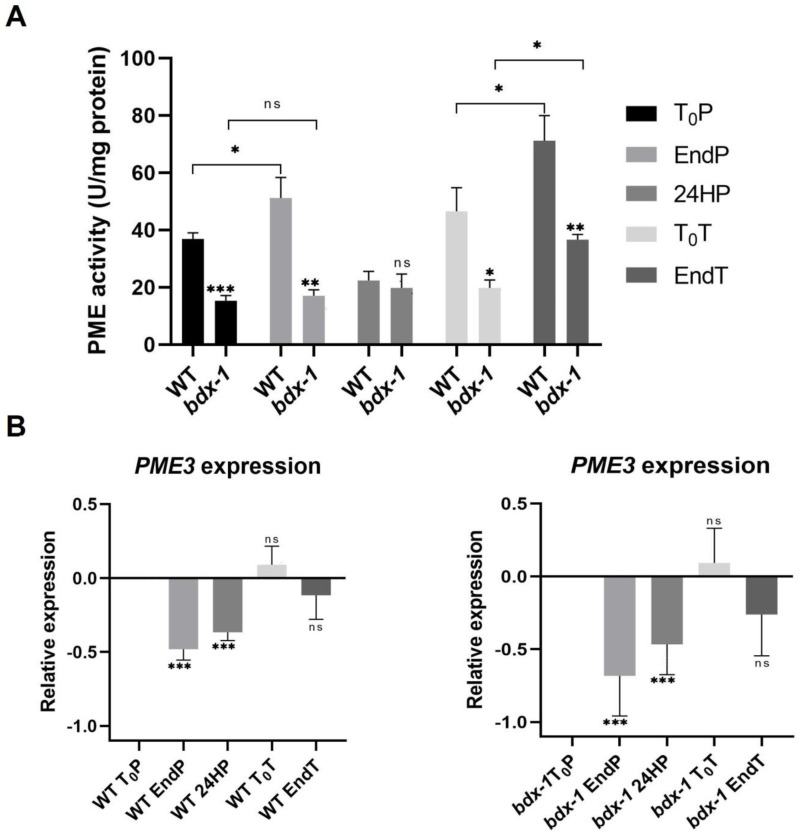
PME activity and *AtPME3* expression of WT and *bdx*-1 lines during thermotolerance and thermomemory. (**A**) Effect of priming and triggering on the total PME activity of WT and *bdx*-1 seedlings. Error bars indicate SEM (n = 4). Statistical analyses were performed using Student’s *t*-test. Asterisks indicate statistically significant differences between WT and *bdx*-1 and between treatments (* *p* < 0.05, ** *p* < 0.01, *** *p* < 0.001). (**B**) Effect of priming and triggering on expression levels of *AtPME3* in WT and *bdx*-1 seedlings. The expression values are relative to T_0_P expression. Error bars indicate S.D. (n = 3). Statistical analyses were performed using Student’s *t*-test. Asterisks indicate statistically significant differences to T_0_P (* *p* < 0.05, ** *p* < 0.01, *** *p* < 0.001, ns, not significant).

The proteins of the DUF642 family participate in different stages of plant development, mainly by increasing the PME activity [11,23]. *In vitro* interaction analyses indicate that a possible target is AtPME3, a PME that is highly expressed in seedlings [22]. Heat stress promotes an increase in total PME activity that is involved in cell wall remodeling, which is crucial for thermotolerance [3]. The total PME activity of T_0_P seedlings from *bdx*-1 corresponded to 50% of the total PME activity of WT seedlings at T_0_P (Figure 4A), although there were no differences in *AtPME3* expression levels. To investigate if the *bdx*-1 heat sensitivity phenotype was related to PME activity modulation, we determined the total PME activity of the WT and *bdx*-1 seedlings during thermopriming and thermotriggering treatments (Figure 4). The difference in PME activity between WT and *bdx*-1 seedlings was observed during both treatments, except for 24 h after priming treatment; the decrease in PME activity detected in WT was similar to the PME activity levels in 24HP seedlings. As it was previously described [13], priming promoted a significant increase in total PME activity in WT seedlings. This increase was not detected in *bdx*-1 seedlings (Figure 4A). As expected, triggering also promoted an increase in PME activity in WT seedlings (Figure 4A). In seedlings, triggering treatment also promoted an increase in PME activity; this activity level was equivalent to the T_0_P seedling from WT. These results suggest that BIIDXI is mainly involved in the increase in PME activity caused by thermopriming.

In general, it has been described that the expression levels of different PME isoforms decrease in response to heat shock or thermopriming treatment [13]. *AtPME3* expressions in response to priming and triggering in WT and *bdx*-1 seedlings exhibit similar dynamics (Figure 4B). The *AtPME3* expression decreased because of priming until after 24 h. At 72 h of treatment, the expression levels had recovered, and the triggering treatment did not affect this expression level.

### 2.4. The HSFA2-Mediated Signaling Pathway Is Activated in bdx-1 Seedlings

**Figure 5:**
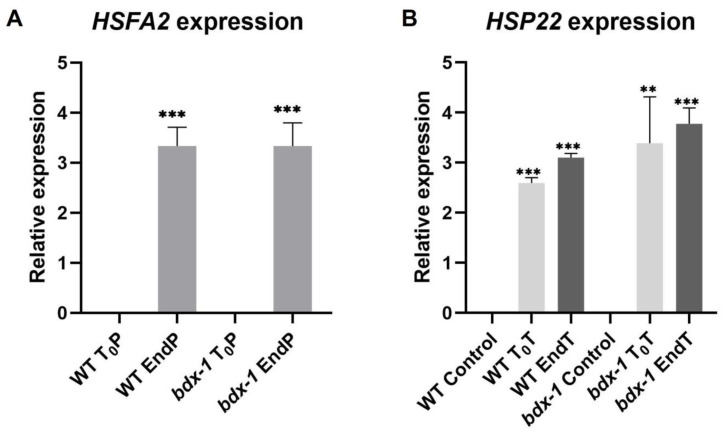
Expression of thermomemory genes in the *bdx*-1 line. (**A**) *HSFA2* expression at the end of priming compared to T_0_P in each line. The expression level is similar in WT and *bdx*-1. (**B**) *HSP22* expression at the beginning and the end of triggering, compared with plants without treatment. For *HSFA2*, the expression values are relative to T_0_P expression and for *HSP22*, to WT control. Error bars indicate S.D. (n = 3). Statistical analyses were performed using Student’s *t*-test. Asterisks indicate statistically significant differences to T_0_P and to WT control (** *p* < 0.01, *** *p* < 0.001).

It has been proposed that PMEs and consequently, cell wall remodeling in response to heat shock, could activate the signaling pathway involved in heat shock response [3]; thus, we determined the expressions of *HSFA2* and *HSP22* during priming and triggering treatments on the WT and *bdx*-1 lines. *HSFA2* expression increased markedly in both lines in response to priming (Figure 5A). *HSP22* expression increased in response to priming in both lines and remained at high levels up to 72 h after treatment. Triggering slightly promoted expressions in the WT and *bdx*-1 lines (Figure 5B). 

## 3. Discussion

The cell walls of different plant cells have complex and dynamic structures that actively participate in the perception of environmental conditions and in the promotion of cellular responses [1]. Pectin modifications could be involved in the response to different abiotic stresses such as salinity, osmostic, heat, and cold, although the mechanisms involved in perceiving the cell wall damage are not yet known. It has been proposed that salt and heat stresses influence the mechanical properties of the cell wall, modifying the pectin degree of esterification [27]. Recently, Gigli–Bisceglia et al. [4] reported that PME activation by salt is a requirement for the activation of salt-induced responses. In our study, we focused on investigating the function of the DUF642 protein BIIDXI during thermotolerance acquisition and in thermomemory. *BIIDXI* gene expression decreased when the seedlings were subjected to 37 °C and to 44 °C during the priming treatment (Figure 1B). In tissues of different plants, it has been determined that heat shock inhibits the gene expression of the DUF642 family [27,31,32]. *BIIDXI* expression increased 24 h after priming (Figure 1C). This increase in expression, unaffected by the second heat shock, could be regulated by HSFA2, a heat stress response factor involved in maintaining a high expression of genes, over several days; it could be involved in thermomemory [33]. The promoter region of *BIIDXI* presents several regulatory regions related to the response to heat stress, and the positive regulation of the expression of a gene of the DUF642 family by HSFA2 has been previously described in tomato [6].

BIIDXI participates in seed development and germination by modifying the pectin degree of esterification by promoting PME activity. An abnormal embryo elongation, resulting from a high methyl esterification of pectins in the cell wall of endosperm cells, was observed in approximately 40% of *bdx*-1 seeds. These misshapen seeds present altered germination and seedling development [24,25]. For this study, we used seedlings from normal seeds. These *bdx*-1 seedlings presented a high sensitivity to the priming treatment, determined by a higher percentage of seedlings with cotyledon bleaching and by the retarded growth of the main root (Figure 2 and Figure 3). An increase in the testa rupture rate was observed in *OEBDX* seeds, but seedling development was not affected. No difference in heat sensitivity was observed (Figure 2). The demethyl esterification rate of PME activity was increased substantially with increasing temperature, but the mechanism for temperature activation is less understood [3]. WT seedlings showed an increase in PME activity because of priming, as previously described (Figure 4; [13]), while at 24 h after treatment, the activity dropped considerably to similar levels determined in T_0_P seedlings from the *bdx*-1 mutant. This decrease in PME activity observed in WT seedlings could be related to the decrease in *BIIDXI* expression resulting from priming treatment (Figure 1B). *bdx*-1 seedlings did not show any changes in PME activity during priming; the PME activity peak present in WT seedlings was not observed (Figure 4). Heat shock also causes decreases in the expressions of different PMEs. In winter oilseed rape, heat shock induced a nearly 10-fold reduction in *PME35* gene expression [34]. In Arabidopsis, the expressions of *AtPM34*, *AtPM28*, and *AtPM7* were reduced at the end of the priming treatment but recovered after 3 h [13]. In the current study, priming promoted a decrease in the expression of *AtPME3*, the PME isoform that interacts *in vitro* with BIIDXI, in both WT and *bdx*-1 seedlings (Figure 4). Huang et al. [13] suggested that a regulator of PME activity might be involved in the increased activity during the response to heat stress. Our results support the hypothesis that BIIDXI is involved in the acquisition of thermotolerance by promoting an increase in PME activity. Furthermore, the mechanism by which BIIDXI participates in the response to heat is through promoting PME activity; this was supported by the identification of the thermotolerance impairment of *Arabidopsis bdx*-1 mutant plants, which was independent from the expression of heat stress-responsive genes. The gene expression patterns of heat-responsive genes in *bdx*-1, such as *HSFA2* and *HSP22*, were shown to be similar to those of WT plants (Figure 5).

In response to triggering, PME activity was significantly increased in WT seedlings, while *AtPME3* and *BIIDXI* expression levels were not affected (Figure 4). These results suggest that BIIDXI could also be involved in thermomemory by promoting cell wall remodeling. The relevance of cell wall remodeling in thermomemory is also suggested by the HSFA2 upregulation of the expression of a high number of genes involved in cell wall dynamics, including several PMEs [6]. However, triggering also promoted a PME activity increase in *bdx*-1, suggesting that in thermomemory, there could be alternate mechanisms to promote PME activation in the absence of BIIDXI. These results can be partially explained by the maintenance of *AtPME3* expression levels during triggering treatment (Figure 4).

The participation of cell wall proteins of the DUF642 family in plant development, especially in the response to stress, has been poorly studied. However, studies in *Arabidopsis* and rice indicate that this family of proteins actively participates in increasing the degree of demethyl esterification of pectins in the cell wall of different cell types by promoting PME activity, although the biochemical mechanism has not been described. The results obtained suggest that BIIDXI participates in the response to heat stress by the activation of PMEs, a process necessary for the acquisition of thermotolerance.

## 4. Methods

### 4.1. Plant Growth Conditions

*Arabidopsis thaliana* seeds of the WT (Col-0), *bdx*-1, and *OEBDX* lines were sown on sterile Murashige and Skoog (MS) medium with 1% sucrose and a pH of 5.7. Seeds were stratified at 4 °C for 48 h and subsequently placed in a CONVIRON growth chamber (Winnipeg, Canada) at 22 °C under long photoperiod conditions (16 h light; 8 h dark). 

#### Transgenic Plants

*bdx*-1: 142260 SALK line with the T-DNA insertion at the end of the second exon of *BIIDXI.* In the homozygous line, the expression of *BIIDXI* was approximately reduced to a 60% [24].

*OEBDX*: Transgenic plants were transformed with a pBIN vector that contained the full-length coding *BIIDXI* sequence under the control of the cauliflower mosaic virus 35S promoter [24]. Expression of *BIIDXI* was highly increased (1000-fold change) in the homozygous transgenic lines [35].

### 4.2. Thermotolerance and Thermomemory Analyses

Thermopriming and thermotriggering treatments were performed based on Olas et al., [29] and is briefly described as follows: Seedlings with two full leaves were subjected to priming stimulus at 6 h after dawn (90 min at 37 °C in a water bath; recovery at 22 °C for 90 min in the growth chamber; 45 min at 44 °C in a water bath), then returned to normal growth conditions (22 °C) for 72 h of the recovery phase, and then subjected to the triggering treatment (45 min at 44 °C) (Figure 1A). To have enough material for subsequent analyses, the treatment time was reduced to promote seedling survival, especially for *bdx*-1 seedlings.

Whole seedling samples were collected and frozen immediately at the beginning and at the end of priming (T_0_P and EndP), 24 h after the end of priming (24HP), 72 h after priming/start of triggering (T_0_T), at the end of triggering (EndT), and 4 days after triggering (4dpT).

The analysis of the percentage of seedlings with cotyledon bleaching in the lines WT, *bdx*-1, and *OEBDX* after priming and triggering was carried out in three independent biological replicates with 10–12 seedlings. The effects of priming on root lengths were also performed in the lines WT, *bdx*-1, and *OEBDX* in three independent biological replicates with 30 seedlings.

All the data analyses were performed using GraphPad Prism version 8.0.0 for windows, Graph Pad software, San Diego, CA, USA, www.graphpad.com.

### 4.3. RNA Isolation, cDNA Synthesis, and Quantitative RT-PCR

Total RNA was extracted from 50 mg of collected tissue at each thermomemory treatment point of WT and *bdx*-1 plants using the TRIzol extraction technique (Invitrogen) according to the manufacturer’s recommendations. RNA was quantified by means of a NanoDrop Lite spectrophotometer (Thermo Fisher Scientific). The cDNA synthesis was performed from 200 ng of total RNA using the Im Prom-II Reverse Transcription System kit (Promega Corporation). The qRT-PCR measurements were performed using SYBR Green Master Mix and detected with an Applied Biosystems StepOne platform (Applied Biosystems). Three or four independent biological replicates with three technical replicates were performed, and either *TUB2* or *ACT7* was used as endogenous controls using the primers listed in Table S1. The specificity of *BIIDXI* primers was confirmed by cloning and sequencing different clones [24]. In silico analysis also confirmed the primer specificity (Table S2).

Expression analyses were performed using the method described by [36]. Student’s *t*-tests were performed using GraphPad Prism version 8.0.0 for windows, Graph Pad software, San Diego, CA, USA, www.graphpad.com.

### 4.4. Determination of Pectin Methyl Esterase Activity

The assay was performed based on Huang et al., [13]. Seedlings samples (200 mg) were ground and resuspended in phosphate–citrate buffer (0.1 M citric acid; Na_2_HPO_4_ 0.2 M; NaCl 1 M; pH 5) at 4 °C, with a 3:1 ratio. Then, the samples were centrifuged at 12,000 rpm at 4 °C, the supernatants were recovered, and the total proteins were quantified with the Bradford method.

For the PME activity analysis, 5 µg of protein was added to 1 mL of 0.1% esterified pectin (SigmaAldrich) in 50 mM phosphate buffer (Na_2_HPO_4_ [0.2 M]; NaH_2_PO_4_ [0.2 M]; pH 7) and incubated at 37 °C for 1 h. At the end of the incubation, 200 µL of 0.02% ruthenium red was added and incubated at room temperature for 15 min. Finally, 200 µL of 0.6 M CaCl_2_ was added and centrifuged at 12,000 rpm for 10 min. Supernatant absorbance at 534 nm was recorded. Four independent biological replicates for each sample, with three technical replicates, were performed. The activity of commercially purifying PME (SigmaAldrich) was performed using the protocol previously described. The standard curve was used to calculate the specific activity for each replicate (U/mg protein). Student’s *t*-tests were performed using GraphPad Prism version 8.0.0 for windows, Graph Pad software, San Diego, CA, USA, www.graphpad.com.

## Supplementary Materials

The following supporting information can be downloaded at: https://www.mdpi.com/article/10.3390/plants11223049/s1, Figure S1: *At3g08030* and *DGR2* (*At5g25460*) gene expression in WT Col. during thermopriming and thermo-triggering treatments. Table S1: List of primers used for qRT-PCR experiment. Table S2: Alignment of *BIIDXI* primers to DUF642 gene sequences of *Arabidopsis thaliana.*

## References

[1] Wolf S. (2022). Cell Wall Signaling in Plant Development and Defense. Annu. Rev. Plant Biol..

[2] Balazadeh S. (2022). A ‘hot’ cocktail: The multiple layers of thermomemory in plants. Curr. Opin. Plant Biol..

[3] Wu H.-C., Bulgakov V.P., Jinn T.-L. (2018). Pectin methylesterases: Cell wall remodeling proteins are required for plant response to heat stress. Front. Plant Sci..

[4] Gigli-Bisceglia N., Van Zelm E., Huo W., Lamers J., Testerink C. (2022). Arabidopsis root responses to salinity depend on pectin modification and cell wall sensing. Development.

[5] Fabri J.H.T.M., Rocha M.C., Fernandes C.M., Persinoti G.F., Ries L.N.A., Cunha A.F.D., Goldman G.H., Del Poeta M., Malavazi I. (2021). The heat shock transcription factor HsfA is essential for thermotolerance and regulates cell wall integrity in *Aspergillus fumigatus*. Front. Microbiol..

[6] Fragkostefanakis S., Mesihovic A., Simm S., Paupière M.J., Hu Y., Paul P., Mishra S.K., Tschiersch B., Theres K., Bovy A. (2016). HsfA2 controls the activity of developmentally and stress-regulated heat stress protection mechanisms in tomato male reproductive tissues. Plant Physiol..

[7] Novaković L., Guo T., Bacic A., Sampathkumar A., Johnson K.L. (2018). Hitting the wall—Sensing and signaling pathways involved in plant cell wall remodeling in response to abiotic stress. Plants.

[8] Wolf S., Mouille G., Pelloux J. (2009). Homogalacturonan methyl-esterification and plant development. Mol. Plant.

[9] Willats W.G.T., Orfila C., Limberg G., Buchholti H.C., van Alebeek G.J.W.M., Voragen A.G.J., Marcus S.E., Mikkelsen J.D., Murray B.S., Knox J.P. (2001). Modulation of the degree and pattern of methyl-esterification of pectic homogalacturonan in plant cell walls: Implications for pectin methyl esterase action, matrix properties, and cell adhesion. J. Biol. Chem..

[10] Wormit A., Usadel B. (2018). The multifaceted role of pectin methylesterase inhibitors (PMEIs). Int. J. Mol. Sci..

[11] Cruz-Valderrama J.E., Gómez-Maqueo X., Salazar-Iribe A., Zúñiga-Sánchez E., Hernández-Barrera A., Quezada-Rodríguez E., Gamboa-deBuen A. (2019). Overview of the role of cell wall DUF642 proteins in plant development. Int. J. Mol. Sci..

[12] Pelloux J., Rusterucci C., Mellerowicz E.J. (2007). New insights into pectin methylesterase structure and function. Trends Plant Sci..

[13] Huang Y.-C., Wu H.C., Wang Y.D., Liu C.H., Lin C.C., Luo D.L., Jinn T.L. (2017). PECTIN METHYLESTERASE34 contributes to heat tolerance through its role in promoting stomatal movement. Plant Physiol..

[14] Wu H.C., Yu S.Y., Wang Y.D., Jinn T.L. (2022). Guard Cell-Specific Pectin METHYLESTERASE53 Is Required for Abscisic Acid-Mediated Stomatal Function and Heat Response in Arabidopsis. Front. Plant Sci..

[15] Wu H.C., Hsu S.F., Luo D.L., Chen S.J., Huang W.D., Lur H.S., Jinn T.L. (2010). Recovery of heat shock-triggered released apoplastic Ca2+ accompanied by pectin methylesterase activity is required for thermotolerance in soybean seedlings. J. Exp. Bot..

[16] Bosch M., Hepler P.K. (2005). Pectin methylesterases and pectin dynamics in pollen tubes. Plant Cell.

[17] Balestrieri C., Castaldo D., Giovane A., Quagliuolo L., Servillo L. (1990). A glycoprotein inhibitor of pectin methylesterase in kiwi fruit (*Actinidia chinensis*). Eur. J. Biochem..

[18] Coculo D., Lionetti V. (2022). The Plant Invertase/Pectin Methylesterase Inhibitor Superfamily. Front. Plant Sci..

[19] Chen J., Chen X., Zhang Q., Zhang Y., Ou X., An L., Feng H., Zhao Z. (2018). A cold-induced pectin methyl-esterase inhibitor gene contributes negatively to freezing tolerance but positively to salt tolerance in Arabidopsis. J. Plant Physiol..

[20] Vázquez-Lobo A., Roujol D., Zuñiga-Sánchez E., Albenne C., Piñero D., de Buen A.G., Jamet E. (2012). The highly conserved spermatophyte cell wall DUF642 protein family: Phylogeny and first evidence of interaction with cell wall polysaccharides in vitro. Mol. Phylogenetics Evol..

[21] Jamet E., Canut H., Boudart G., Pont-Lezica R.F. (2006). Cell wall proteins: A new insight through proteomics. Trends Plant Sci..

[22] Zúñiga-Sánchez E., Gamboa-de Buen A. (2012). The two DUF642 At5g11420 and At4g32460-encoded proteins interact in vitro with the AtPME3 catalytic domain. Protein Interact..

[23] Wang M., Zhu X., Peng G., Liu M., Zhang S., Chen M., Liao S., Wei X., Xu P., Tan X. (2022). Methylesterification of cell-wall pectin controls the diurnal flower-opening times in rice. Mol. Plant.

[24] Zúñiga-Sánchez E., Soriano D., Martínez-Barajas E., Orozco-Segovia A., Gamboa-deBuen A. (2014). *BIIDXI*, the *At4g32460* DUF642 gene, is involved in pectin methyl esterase regulation during *Arabidopsis thaliana* seed germination and plant development. BMC Plant Biol..

[25] Cruz-Valderrama J.E., Jiménez-Durán K., Zúñiga-Sánchez E., Salazar-Iribe A., Márquez-Guzmán J., Gamboa-deBuen A. (2018). Degree of pectin methyl esterification in endosperm cell walls is involved in embryo bending in *Arabidopsis thaliana*. Biochem. Biophys. Res. Commun..

[26] Salazar-Iribe A., Agredano-Moreno L.T., Zúñiga-Sánchez E., Jiménez-Garcia L.F., Gamboa-deBuen A. (2016). The cell wall DUF642 At2g41800 (TEB) protein is involved in hypocotyl cell elongation. Plant Sci..

[27] Shin Y., Chane A., Jung M., Lee Y. (2021). Recent advances in understanding the roles of pectin as an active participant in plant signaling networks. Plants.

[28] Pinski A., Betekhtin A., Skupien-Rabian B., Jankowska U., Jamet E., Hasterok R. (2021). Changes in the cell wall proteome of leaves in response to high temperature stress in *Brachypodium distachyon*. Int. J. Mol. Sci..

[29] Olas J.J., Apelt F., Annunziata M.G., John S., Richard S.I., Gupta S., Kragler F., Balazadeh S., Mueller-Roeber B. (2021). Primary carbohydrate metabolism genes participate in heat-stress memory at the shoot apical meristem of *Arabidopsis thaliana*. Mol. Plant.

[30] Liu J., Liu Y., Wang S., Cui Y., Yan D. (2022). Heat Stress Reduces Root Meristem Size via Induction of Plasmodesmal Callose Accumulation Inhibiting Phloem Unloading in Arabidopsis. Int. J. Mol. Sci..

[31] Bustamante C.A., Budde C.O., Borsani J., Lombardo V.A., Lauxmann M.A., Andreo C.S., Lara M.V., Drincovich M.F. (2012). Heat treatment of peach fruit: Modifications in the extracellular compartment and identification of novel extracellular proteins. Plant Physiol. Biochem..

[32] Ngcala M.G., Goche T., Brown A.P., Chivasa S., Ngara R. (2020). Heat stress triggers differential protein accumulation in the extracellular matrix of sorghum cell suspension cultures. Proteomes.

[33] Lämke J., Brzezinka K., Altmann S., Bäurle I. (2016). A hit-and-run heat shock factor governs sustained histone methylation and transcriptional stress memory. EMBO J..

[34] Yu E., Fan C., Yang Q., Li X., Wan B., Dong Y., Wang X., Zhou Y. (2014). Identification of heat responsive genes in *Brassica napus* siliques at the seed-filling stage through transcriptional profiling. PLoS ONE.

[35] Salazar-Iribe A., Cruz-Valderrama J.E., Jímenez-Durán K., Gómez-Maqueo X., Gamboa-deBuen A. (2018). BIIDXI, a DUF642 cell wall protein, is involved in hypocotyl growth via auxin efflux. J. Plant. Physiol..

[36] Willems E., Leyns L., Vandesompele J. (2008). Standardization of real-time PCR gene expression data from independent biological replicates. Anal. Biochem..

