# Supplementary material for: BIIDXI, a DUF642 Cell Wall Protein That Regulates Pectin Methyl Esterase Activity, Is Involved in Thermotolerance Processes in Arabidopsis thaliana
Source: Plants (Basel). 2022 Nov 11;11(22):3049. doi: 10.3390/plants11223049 (PMC9694414; doi:10.3390/plants11223049)
Supplement: Supplementary file 1 [file plants-11-03049-s001.zip › plants-1993989-supplementary.pdf]

BIIDXI, a DUF642 CellWall Protein That Regulates Pectin Methyl Esterase Activity, Is Involved in Thermotolerance Processes in *Arabidopsis thaliana*

Eduardo Pineda-Hernández[1], José Erik Cruz-Valderrama[1], Ximena Gómez-Maqueo[1], Eleazar Martínez-Barajas[2] and Alicia Gamboa-deBuen[1,]*

[1] Instituto de Ecología, Universidad Nacional Autónoma de México (UNAM), Ciudad de México 04510, Mexico

[2] Facultad de Química, Universidad Nacional Autónoma de México (UNAM), Ciudad de México 04510, Mexico

***** Correspondence: agamboa@ecologia.unam.mx

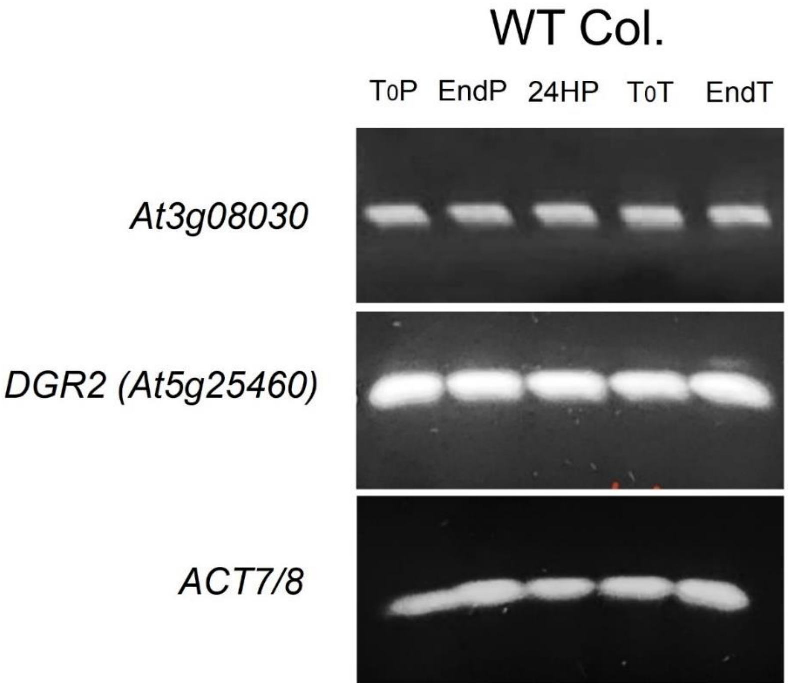

Figure S1: *At3g08030* and *DGR2* (*At5g25460*) gene expression in WT Col. during thermopriming and thermotriggering treatments. The expression of these two DUF642 genes is not changed in response to heat.

Table S1: List of primers used for qRT-PCR experiments

**Genes**	**Primer Forward**	**Primer Reverse**
TUB2 ***(At5g62690)***	5’-GAGCCTTACAACGCTACTCTGTCTGTC-3’	5’ACACCAGACATAGTAGCAGAAATC AAG-3’
Actina 7/8 ***(At5g09810)/(At1g49240)***	5´-GGTCGTACAACCGGTATTGT-3’	5´-GATAGCATGTGGAAGTGAGAA-3’
BDX ***(At3g32460)***	5’-CTCTCGCTCACTCTTCTCCAA-3’	5’ CGACAAAGCCTGAGAGTTCCC-3’
PME3 ***(At3g14310)***	5’-TCAAATGTCTCATCGCCGG-3’	5’AGCGTGGATGTCACAGTC -3’
HSFA2 ***(At2g26150)***	5’-GCAGCGTTGGATGTGAAAGTGG-3’	5’-TTGGCTGTCCCAATCCAAAGGC-3’
HSP22 (At4g10250)	5´-CGGTTCCCTGATCCATTCAA-3’	5´-GCCCTTCTGCTGTTTCTTTCC-3’

Table S2. Alignment of *BIIDXI* primers to DUF642 genes.

Results for linear 1591 residue sequence "AT1G29980.1 | Symbols: no symbol available | no full name available | chr1:10503239-10

```
   1   AAAAAAGAAGAAAAAAAAAAGACAAGGAGTGAGGAATCTTGGATAAAAGGACACTAACCTTCCCTGTCTTTGCTCGCTGCCCGAAAAGAACATACAGAAAGACACT
   1          10         20         30         40         50         60         70         80         90        100
   1   TTTTTTCTTCTTTTTTTTTTCTGTTCCTCACTCCTTAGAACCTATTTTCCTGTGATTGGAAGGGACAGAAACGAGCGACGGGCTTTTCTTGTATGTCTTTCTGTGA
 106    TTCACATTTCACACTTACAGAGAGAGAGAAGAGAGATAAAGAGAGCATTTAGATATCTTGAGGAAGACGAAGAAGAAGTAGAGGAGAGATATGGTTCCGAGCAAC
 106  110  120  130  140  150  160  170  180  190                                                     200
 106   AAGTGTAAAGTGTGAATGTCTCTCTCTCTTCTCTCTATTTCTCTCGTAAATCTATAGAACTCCTTCTGCTTCTTCTTCATCTCCTCTCTATACCAAGGCTCGTTG
 211    AATAGATGCAAATGGAGTTCCATTTTCTTGTTTCTGCTCAGTGTCTCCGTTGCCGTCTTAGTCGCCGTCGCTGACGACAAGTCTCCGGCGGTTGAGGATGGTTTA
 211  220  230  240  250  260  270  280  290  300                                                     310
 211   TTATCTACGTTTACCTCAAGGTAAAAGAACAAAGACGAGTCACAGAGGCAACGGCAGAATCAGCGGCAGCGACTGCTGTTCAGAGGCCGCCAACTCCTACCAAAT
 316    GTGATCAACGGCGACTTCGAAACATCACCGTCAAGTGGCTTTCCTGACGACGGAGTAACTGACGGACCATCCGATATCCCAAGCTGGAAATCCAACGGTACGGTG
 316  320  330  340  350  360  370  380  390  400                                                     410
 316   CACTAGTTGCCGCTGAAGCTTTGTAGTGGCAGTTCACCGAAAGGACTGCTGCCTCATTGACTGCCTGGTAGGCTATAGGGTTCGACCTTTAGGTTGCCATGCCAC
 421    GAGCTAATAAATTCCGGTCAAAAACAAGGCGGAATGATCCTCATTGTCCCACAAGGCCGTCACGCCGTCCGATTAGGGAACGACGCAGAGATCAGCCAAGATCTA
 421  430  440  450  460  470  480  490  500  510                                                     520
 421   CTCGATTATTTAAGGCCAGTTTTTGTTCCGCCTTACTAGGAGTAACAGGGTGTTCCGGCAGTGCGGCAGGCTAATCCCTTGCTGCGTCTCTAGTCGGTTCTAGAT
 526    ACGGTTGAGAAAGGTTTTGTCTATTCAGTCACGTTCAGCGCTGCTCGCACGTGCGCTCAACTCGAATCGATAAACGTGTCGGTAGCGTCTGTGAACGCAGACGCG
 526  530  540  550  560  570  580  590  600  610                                                     620
 526    TGCCAACTCTTTCCAAAACAGATAAGTCAGTGCAAGTCGCGACGAGCGTGCACGCGAGTTGAGCTTAGCTATTTGCACAGCCATCGCAGACACTTGCGTCTGCGC
 631    GACGATATGCTGGCGTCGCGGAACGTTGATTTGCAAACGCTTTATAGCGTTCAAGGGTGGGACCCTTATGCGTGGGCGTTTGAAGCGGAAGATGATCATGTACGG
 631  640  650  660  670  680  690  700  710  720                                                     730
 631    CTGCTATACGACCGCAGCGCCTTGCAACTAAACGTTTGCGAAATATCGCAAGTTCCCACCCTGGGAATACGCACCCGCAAACTTCGCCTTCTACTAGTACATGCC
 736    TTGGTTTTTAAGAATCCGGGTATGGAAGATGATCCGACTTGTGGACCAATCATCGATGACATTGCTATTAAGAAGCTCTTTACTCCTGATAAACCCAAAGATAAT
 736     740        750        760        770        780        790        800        810        820        830
 736    AACCAAAAATTCTTAGGCCCATACCTTCTACTAGGCTGAACACCTGGTTAGTAGCTACTGTAACGATAATTCTTCGAGAAATGAGGACTATTTGGGTTTCTATTA
 841    GCGGTGATTAATGGAGATTTCGAAGATGGTCCATGGATGTTTAGGAACACGTCTCTTGGTGTTTTACTTCCGACAAACCTCGACGAAGAAATCTCGTCTCTTCCT
 841          850        860        870        880        890        900        910        920        930        940
 841    CGCCACTAATTACCTCTAAAGCTTCTACCAGGTACCTACAAATCCTTGTGCAGAGAACCACAAAATGAAGGCTGTTTGGAGCTGCTTCTTTAGAGCAGAGAAGGA
 946    GGTTGGACTGTTGAATCGAACAGAGCGGTTCGGTTTGTTGACTCGGATCACTTCTCTGTTCCAAAGGGCAAACGAGCCGTCGAGCTACTCTCAGGCAAAGAAGGA
 946     950        960        970        980        990       1000       1010       1020       1030       1040
 946    CCAACCTGACAACTTAGCTTGTCTCGCCAAGCCAAACAACTGAGCCTAGTGAAGAGACAAGGTTTCCCGTTTGCTCGGCAGCTCGATGAGAGTCCGTTTCTTCCT
1051    ATTATTTCACAAATGGTTGAGACCAAAGCAGATAAGCCGTACATATTGTCTTTCTCACTAGGCCACGCAGGTGATAAATGCAAGGAACCATTGGCTATTATGGCA
1051         1060       1070       1080       1090       1100       1110       1120       1130       1140       1150
1051    TAATAAAGTGTTTACCAACTCTGGTTTCGTCTATTCGGCATGTATAACAGAAAGAGTGATCCGGTGCGTCCACTATTTACGTTCCTTGGTAACCGATAATACCGT
1156    TTCGCAGGTGATCAGGCGCAGAACTTTCATTACATGGCGCAAGCGAACTCCAGTTTCGAAAAGGCCGGTTTGAATTTCACGGCTAAGGCTGATCGAACCAGAGTT
1156     1160       1170       1180       1190       1200       1210       1220       1230       1240       1250
1156    AAGCGTCCACTAGTCCGCGTCTTGAAAGTAATGTACCGCGTTCGCTTGAGGTCAAAGCTTTTCCGGCCAAACTTAAAGTGCCGATTCCGACTAGCTTGGTCTCAA
1261    GCGTTCTACAGTGTTTATTACAATACGAGGACTGATGATATGAGCTCCTTGTGTGGACCTGTGATCGATGACGTTAGAGTGTGGTTCTCCGGGTCGAAGAGAATC
1261         1270       1280       1290       1300       1310       1320       1330       1340       1350       1360
1261    CGCAAGATGTCACAAATAATGTTATGCTCCTGACTACTATACTCGAGGAACACACCTGGACACTAGCTACTGCAATCTCACACCAAGAGGCCCAGCTTCTCTTAG
1366    GGAGCTGGGTTTGGGTTTTGGGTTTTTGTTCTTCTCGTTGTCGGTTTGGTTTAAGTAACTCAAAATTTAGATGTTCCGGTTTTATTCTAGTGTACCAGTAAAATG
1366     1370       1380       1390       1400       1410       1420       1430       1440       1450       1460
1366    CCTCGACCCAAACCCAAAACCCAAAAACAAGAAGAGCAACAGCCAAACCAAATTCATTGAGTTTTAAATCTACAAGGCCAAAATAAGATCACATGGTCATTTTAC
1471    TTGTTGCAAACCAAGAAGAATTCTGTATGGTTTATTTATCCATCGGGTTAAAGCAAATTCGAAGGTGTTTATCGTTATGCAAAAGCTTGTCCCAACGAAAATCGA
1471         1480       1490       1500       1510       1520       1530       1540       1550       1560       1570
1471   AACAACGTTTGGTTCTTCTTAAGACATACCAAATAAATAGGTAGCCCAATTTCGTTTAAGCTTCCACAAATAGCAATACGTTTTCGAACAGGGTTGCTTTTAGCT
1576 GAGTTTTTTTCTATAG
1576     1580       1590
1576 CTCAAAAAAAGATATC
```

Primer:	Sequence:
BDXForward	5'-CTCTCGCTCACTCTTCTCCAA-3'
BDXReverse	5'-CGACAAAGCCTGAGAGTTCCC-3'

Results for linear 1586 residue sequence "AT1G80240.1 | Symbols: DGR1 | DUF642 L-GalL responsive gene 1 | chr1:30171357-30173109

```
   1   GAATAATTTAGATAGGCAAACGCAAATTACGTTTGAATATATAGTTTAATATAATGGTTGTTAAGGCAGCCCCTTAAACAATACATTGAAGAAATGGAAATGCTA
   1          10         20         30         40         50         60         70         80         90        100
   1   CTTATTAAATCTATCCGTTTGCGTTTAATGCAAACTTATATATCAAATTATATTACCAACAATTCCGTCGGGGAATTTGTTATGTAACTTCTTTACCTTTACGAT
 106    ATCATGAAGTCAAATGTTTCCTGAGCTAGCTGTCTGTGTCTGCGTGGCACCTGACAATAGAACCCAGGATGCTTCCAGTGGCATTCCCGTTGTTTTCTTCAAAAC
 106     110        120        130        140        150        160        170        180        190        200
 106   TAGTACTTCAGTTTACAAAGGACTCGATCGACAGACACAGACGCACCGTGGACTGTTATCTTGGGTCCTACGAAGGTCACCGTAAGGGCAACAAAAGAAGTTTTG
 211    CAGTGCTCGTCTTCTATTCTCAAACTCATAAAAACCCAGCCTTTCTTCACCCTTTGGATAGTACTCACCATCTGATCTTCATCTATCACCCAAGGAAGTCATGAT
 211          220        230        240        250        260        270        280        290        300        310
 211   GTCACGAGCAGAAGATAAGAGTTTGAGTATTTTTGGGTCGGAAAGAAGTGGGAAACCTATCATGAGTGGTAGACTAGAAGTAGATAGTGGGTTCCTTCAGTACTA
 316    GTACCAAGAAGCAGCACTCCTCTTAGCTCTCCTCTTCATCTCTTCGAATGTCGTCTTATCGGCCCCAGTCCGCGATGGTTTATTACCAAACGGCAACTTCGAGTT
 316     320        330        340        350        360        370        380        390        400        410
 316   CATGGTTCTTCGTCGTGAGGAGAATCGAGAGGAGAAGTAGAGAAGCTTACAGCAGAATAGCCGGGGTCAGGCGCTACCAAATAATGGTTTGCCGTTGAAGCTCAA
 421    GGGTCCTAAACCCTCCCAAATGAAAGGATCCGTGGTTAAGGAACGAACCGCCGTACCAAACTGGAACATAATCGGCTTTGTGGAGTTCATTAAATCCGGTCAGAA
 421          430        440        450        460        470        480        490        500        510        520
 421   CCCAGGATTTGGGAGGGTTTACTTTCCTAGGCACCAATTCCTTGCTTGGCGGCATGGTTTGACCTTGTATTAGCCGAAACACCTCAAGTAATTTAGGCCAGTCTT
 526    ACAAGATGACATGGTCTTGGTCGTACCGCAGGGTTCCTCCGCCGTTAGACTAGGCAACGAAGCCTCAATCTCCCAGAAAATATCCGTCCTACCTGGCCGTCTGTA
 526     530        540        550        560        570        580        590        600        610        620
 526    TGTTCTACTGTACCAGAACCAGCATGGCGTCCCAAGGAGGCGGCAATCTGATCCGTTGCTTCGGAGTTAGAGGGTCTTTTATAGGCAGGATGGACCGGCAGACAT
 631    CTCAATAACGTTCAGCGCCGCTCGAACCTGCGCTCAAGACGAGAGGCTCAATATATCCGTGACGCATGAGTCAGGTGTGATCCCTATCCAGACGATGTACGGCAG
 631          640        650        660        670        680        690        700        710        720        730
 631    GAGTTATTGCAAGTCGCGGCGAGCTTGGACGCGAGTTCTGCTCTCCGAGTTATATAGGCACTGCGTACTCAGTCCACACTAGGGATAGGTCTGCTACATGCCGTC
 736    CGATGGTTGGGACTCATACTCATGGGCTTTTAAAGCTGGTGGTCCCGAAATCGAGATCCGGTTCCATAACCCTGGTGTTGAAGAGCACCCGGCTTGCGGACCTTT
 736     740        750        760        770        780        790        800        810        820        830
 736    GCTACCAACCCTGAGTATGAGTACCCGAAAATTTCGACCACCAGGGCTTTAGCTCTAGGCCAAGGTATTGGGACCACAACTTCTCGTGGGCCGAACGCCTGGAAA
 841    GATCGACGCCGTGGCTATCAAGGCTTTGTTCCCTCCCAGATTCTCTGGATATAATCTGATAAAGAATGGGAATTTCGAAGAAGGACCTTACGTGTTTCCCACGGC
 841          850        860        870        880        890        900        910        920        930        940
 841    CTAGCTGCGGCACCGATAGTTCCGAAACAAGGGAGGGTCTAAGAGACCTATATTAGACTATTTCTTACCCTTAAAGCTTCTTCCTGGAATGCACAAAGGGTGCCG
 946    AAAATGGGGAGTACTGATCCCACCGTTCATCGAGGACGATAACAGCCCATTGCCCGGTTGGATGATCGAGTCTCTCAAAGCCGTTAAGTACGTAGACAAAGCACA
 946     950        960        970        980        990       1000       1010       1020       1030       1040
 946 TTTTACCCCTCATGACTAGGGTGGCAAGTAGCTCCTGCTATTGTCGGGTAACGGGCCAACCTACTAGCTCAGAGAGTTTCGGCAATTCATGCATCTGTTTCGTGT
1051 CTTCGCTGTCCCCGAAGGACACCGAGCCATCGAGCTTGTTGGAGGCAAAGAGAGCGCCATTTCTCAGATAGTGAGGACGTCACTGAACAAATTCTACGCCCTCAC
1051          1060       1070       1080       1090       1100       1110       1120       1130       1140       1150
1051 GAAGCGACAGGGGCTTCCTGTGGCTCGGTAGCTCGAACAACCTCCGTTTCTCTCGCGGTAAAGAGTCTATCACTCCTGCAGTGACTTGTTTAAGATGCGGGAGTG
1156 GTTCAACGTGGGAGACGCCAGAGACGGTTGTGAGGGACCAATGATTGTGGAAGCGTTCGCTGGACAGGGCAAGGTCATGGTGGACTACGCTTCTAAAGGAAAAGG
1156     1160       1170       1180       1190       1200       1210       1220       1230       1240       1250
1156 CAAGTTGCACCCTCTGCGGTCTCTGCCAACACTCCCTGGTTACTAACACCTTCGCAAGCGACCTGTCCCGTTCCAGTACCACCTGATGCGAAGATTTCCTTTTCC
1261 CGGGTTTAGACGTGGGAGGCTTGTGTTCAAGGCAGTGTCGGCCAGGACACGTGTCACTTTCTTGAGCACGTTTTACCACATGAAAAGCGATCACTCTGGCTCGCT
1261          1270       1280       1290       1300       1310       1320       1330       1340       1350       1360
1261 GCCCAAATCTGCACCCTCCGAACACAAGTTCCGTCACAGCCGGTCCTGTGCACAGTGAAAGAACTCGTGCAAAATGGTGTACTTTTCGCTAGTGAGACCGAGCGA
1366 ATGTGGTCCGGTGATCGATGATGTTAGGCTGGTAGCGGTTGGGAAACTCCGAGGATGAAAGTTGGGGTCGTTGACTCATTCGGTTGAATATACTACATTAATGGA
1366     1370       1380       1390       1400       1410       1420       1430       1440       1450       1460
1366 TACACCAGGCCACTAGCTACTACAATCCGACCATCGCCAACCCTTTGAGGCTCCTACTTTCAACCCCAGCAACTGAGTAAGCCAACTTATATGATGTAATTACCT
1471 TAATTGTAATTCATGTTACATAATAAAAATGTAATTTACGAACTAGTCCCACAAAATTTATTAACTAGTTAATCACCGTTTCAACATATATTATATATATAACGGAG
1471          1480       1490       1500       1510       1520       1530       1540       1550       1560       1570
1471 ATTAACATTAAGTACAATGTATTATTTTTACATTAAATGCTTGATCAGGGTGTTTTAAATAATTGATCAATTAGTGGCAAAGTTGTATATAATATATATTGCCTC
1576 TTTCATTTTTT
1576     1580
1576 AAAGTAAAAAA
```

Primer:	Sequence:
BDXForward	5'-CTCTCGCTCACTCTTCTCCAA-3'
BDXReverse	5'-CGACAAAGCCTGAGAGTTCCC-3'

Results for linear 1680 residue sequence "AT2G34510.1 | Symbols: AthB-1 | | chr2:14543894-14546986 REVERSE LENGTH=1680" starting

```
   1   ACGACCACCACGCGGTTTGTGATGAAGAGGGTCCCAAAATGGATACACTTCTCTACCTATTTTTTAACATATCCTACAAAATCTCAATGGTTTTTATTTAGTTCG
   1          10        20        30        40        50        60        70        80        90       100
   1   TGCTGGTGGTGCGCCAAACACTACTTCTCCCAGGGTTTTACCTATGTGAAGAGATGGATAAAAAATTGTATAGGATGTTTTAGAGTTACCAAAAATAAATCAAGC
 106   ATAAAGTATTATTCCGAATGTAAATAGTGGAACAAAAAGAACAGCTGGGAGGATTTTGTTTAAAAGGACACTACACTTCCCAGCTTTCCCTCACTGCCCGAAAAG
 106  110  120  130  140  150  160  170  180  190                                                     200
 106   TATTTCATAATAAGGCTTACATTTATCACCTTGTTTTTCTTGTCGACCCTCCTAAAACAAATTTTCCTGTGATGTGAAGGGTCGAAAGGGAGTGACGGGCTTTTC
 211   AACACTGCAGAAACACTTTCCTCTGTTCACAGAGAAAGAGAGAGATGATGCTTTACAGCAACAATAGTTGGAGATCGAATTCCATTTTAATACTTCTACTTGGTC
 211  220  230  240  250  260  270  280  290  300                                                          310
 211   TTGTGACGTCTTTGTGAAAGGAGACAAGTGTCTCTTTCTCTCTCTACTACGAAATGTCGTTGTTATCAACCTCTAGCTTAAGGTAAAATTATGAAGATGAACCAG
 316   TCTCCATTGTGGCCGCCGCAGACTCCGCCGGCAAAACTTCACCGGTCGAAGACGGCTTGGTGGTTAACGGCGACTTTGAGACACCGCCGTCAAACGGCTTCCCTG
 316  320  330  340  350  360  370  380  390  400                                                     410
 316   AGAGGTAACACCGGCGGCGTCTGAGGCGGCCGTTTTGAAGTGGCCAGCTTCTGCCGAACCACCAATTGCCGCTGAAACTCTGTGGCGGCAGTTTGCCGAAGGGAC
 421   ATGACGCAATCATCGAGGACACCTCCGAGATCCCGAGCTGGCGATCCGATGGTACGGTGGAGCTAATCAAGTCCGGTCAAAAACAAGGCGGGATGATCTTGATCG
 421  430  440  450  460  470  480  490  500  510                                                          520
 421   TACTGCGTTAGTAGCTCCTGTGGAGGCTCTAGGGCTCGACCGCTAGGCTACCATGCCACCTCGATTAGTTCAGGCCAGTTTTTGTTCCGCCCTACTAGAACTAGC
 526   TGCCTGAGGGCCGTCACGCTGTTAGATTAGGAAACGATGCAGAGATCAGCCAAGAACTTACGGTGGAGAAAGGCTCTATCTACTCCGTCACGTTCAGTGCAGCCC
 526  530  540  550  560  570  580  590  600  610                                                     620
 526   ACGGACTCCCGGCAGTGCGACAATCTAATCCTTTGCTACGTCTCTAGTCGGTTCTTGAATGCCACCTCTTTCCGAGATAGATGAGGCAGTGCAAGTCACGTCGGG
 631   GCACATGTGCACAACTCGAGTCGCTGAATGTTTCGGTAGCTTCTTCTGATGAACCTATCGCATCGCAAACCATTGACTTGCAAACGGTGTACAGCGTTCAAGGAT
 631  640  650  660  670  680  690  700  710  720                                                          730
 631   CGTGTACACGTGTTGAGCTCAGCGACTTACAAAGCCATCGAAGAAGACTACTTGGATAGCGTAGCGTTTGGTAACTGAACGTTTGCCACATGTCGCAAGTTCCTA
 736   GGGATCCATATGCATGGGCGTTTGAAGCGGTTGTGGATCGCGTCCGGTTGGTTTTTAAGAACCCTGGCATGGAGGATGATCCTACTTGTGGACCTATCATTGACG
 736    740       750       760       770       780       790       800       810       820       830
 736   CCCTAGGTATACGTACCCGCAAACTTCGCCAACACCTAGCGCAGGCCAACCAAAAATTCTTGGGACCGTACCTCCTACTAGGATGAACACCTGGATAGTAACTGC
 841   ACATTGCCGTTAAGAAGCTCTTTACTCCTGATAAACCCAAAGGCAATGCAGTGATTAATGGAGATTTTGAAGAAGGTCCATGGATGTTTAGGAACACTACCTTAG
 841          850       860       870       880       890       900       910       920       930       940
 841   TGTAACGGCAATTCTTCGAGAAATGAGGACTATTTGGGTTTCCGTTACGTCACTAATTACCTCTAAAACTTCTTCCAGGTACCTACAAATCCTTGTGATGGAATC
 946   GTGTTCTGCTTCCAACAAACCTCGATGAAGAAATATCGTCTCTTCCTGGATGGACCGTCGAATCGAACCGAGCAGTACGGTTTATTGACTCAGACCATTTCTCGG
 946    950       960       970       980       990      1000      1010      1020      1030      1040
 946   CACAAGACGAAGGTTGTTTGGAGCTACTTCTTTATAGCAGAGAAGGACCTACCTGGCAGCTTAGCTTGGCTCGTCATGCCAAATAACTGAGTCTGGTAAAGAGCC
1051   TCCCCGAGGGGAAGCGAGCTTTGGAACTTTTATCGGGCAAAGAAGGCATAATTTCTCAAATGGTTGAGACAAAGGCGAACATTCCGTACAAGATGTCTTTCTCTT
1051         1060      1070      1080      1090      1100      1110      1120      1130      1140      1150
1051   AGGGGCTCCCCTTCGCTCGAAACCTTGAAAATAGCCCGTTTCTTCCGTATTAAAGAGTTTACCAACTCTGTTTCCGCTTGTAAGGCATGTTCTACAGAAAGAGAA
1156   TGGGACACGCAGGGGACAAGTGTAAGGAACCTTTGGCTGTAATGGCTTTTGCTGGAGATCAAGCACAGAACTTTCATTATATGGCGCAAGCAAACTCGAGTTTCG
1156   1160      1170      1180      1190      1200      1210      1220      1230      1240      1250
1156   ACCCTGTGCGTCCCCTGTTCACATTCCTTGGAAACCGACATTACCGAAAACGACCTCTAGTTCGTGTCTTGAAAGTAATATACCGCGTTCGTTTGAGCTCAAAGC
1261   AAAGATCGGAGTTGAACTTCACTGCGAAAGCTGAACGTACGAGGATCGCCTTCTACAGCATTTATTACAATACGAGGACGGACGATATGACTTCATTGTGTGGAC
1261         1270      1280      1290      1300      1310      1320      1330      1340      1350      1360
1261   TTTCTAGCCTCAACTTGAAGTGACGCTTTCGACTTGCATGCTCCTAGCGGAAGATGTCGTAAATAATGTTATGCTCCTGCCTGCTATACTGAAGTAACACACCTG
1366   CTGTGATTGATGACGTTAAGGTTTGGTTCTCCGGGTCTAGTAGAATTGGATTTAGTTTTCCGCTTTTTATTCTTCTTTCTTTGGTTTTCATCTAGATTGTTCCGG
1366   1370      1380      1390      1400      1410      1420      1430      1440      1450      1460
1366   GACACTAACTACTGCAATTCCAAACCAAGAGGCCCAGATCATCTTAACCTAAATCAAAAGGCGAAAAATAAGAAGAAAGAAACCAAAAGTAGATCTAACAAGGCC
1471   TTCAGAAATTGTATTGGTAGACCGGGAATTAAGACGGGATTCCACGTTGTATGATGTATCGTTGTATGATGGATCGGTTCAAGGTACAAGAAATGACAAAGTACT
1471         1480      1490      1500      1510      1520      1530      1540      1550      1560      1570
1471   AAGTCTTTAACATAACCATCTGGCCCTTAATTCTGCCCTAAGGTGCAACATACTACATAGCAACATACTACCTAGCCAAGTTCCATGTTCTTTACTGTTTCATGA
1576   TATGCAATTCATGAGTTTAGAGCAATCATGCTTAAACAATTATCAAACTAGCTAGGCTATACATTTTTGTAGCTTTATTGCAAATTAATGTAAACTAGTTCTTGG
1576   1580      1590      1600      1610      1620      1630      1640      1650      1660      1670
1576   ATACGTTAAGTACTCAAATCTCGTTAGTACGAATTTGTTAATAGTTTGATCGATCCGATATGTAAAAACATCGAAATAACGTTTAATTACATTTGATCAAGAACC
```

Primer:	Sequence:
BDXForward	5'-CTCTCGCTCACTCTTCTCCAA-3'
BDXReverse	5'-CGACAAAGCCTGAGAGTTCCC-3'

Results for linear 1504 residue sequence "AT2G41800.1 | Symbols: TEB | "TEEBE, long in the Mayo-Yoreme language" | chr2:17436443

```
   1   GTTTTGTTGTTGTATATTTCCAAAAATGTACATGCTGCTGCTTCCTCTTTGTTTCTTATTTATAATATCTCATGTCAAGTGATAACTGATAAGTATAAAACCTCC
   1          10         20         30         40         50         60         70         80         90        100

   1   CAAAACAACAACATATAAAGGTTTTTACATGTACGACGACGAAGGAGAAACAAAGAATAAATATTATAGAGTACAGTTCACTATTGACTATTCATATTTTGGAGG
 106    TATATATAGCCCACAAGGTCCTCATTGATATAGGCGAACTAATTAATAAGACTTTAAAATGTCTCTCTATCACTCTCTTAGCATTTTCCTCCTCCTATCTCTCTG
 106     110        120        130        140        150        160        170        180        190        200
 106   ATATATATCGGGTGTTCCAGGAGTAACTATATCCGCTTGATTAATTATTCTGAAATTTTACAGAGAGATAGTGAGAGAATCGTAAAAGGAGGAGGATAGAGAGAC
 211    CCATGGCTCTTACAGTCTTCAACCACGTCGAGTTCCTCATCTAGACGGGATTCTCCCAAATGGAAACTTCGAGATAACCCCTCTCAAATCAAACATGAAGGGTCG
 211          220        230        240        250        260        270        280        290        300        310
 211   GGTACCGAGAATGTCAGAAGTTGGTGCAGCTCAAGGAGTAGATCTGCCCTAAGAGGGTTTACCTTTGAAGCTCTATTGGGGAGAGTTTAGTTTGTACTTCCCAGC
 316    ACAAATAATCGGGGCTAACTCTCTTCCTCATTGGGAAATCGCCGGGCACGTGGAGCTAGTCTCCGGTGGACCTCAGCCTGGAGGATTCTATTTCCCCGTTCCACG
 316     320        330        340        350        360        370        380        390        400        410
 316   TGTTTATTAGCCCCGATTGAGAGAAGGAGTAACCCTTTAGCGGCCCGTGCACCTCGATCAGAGGCCACCTGGAGTCGGACCTCCTAAGATAAAGGGGCAAGGTGC
 421    TGGAGTCCACGCGGTTAGACTCGGAAACTTGGGCACCATCTCTCAGAACGTGAGGGTGAAGAGTGGTTTGGTCTACTCTCTAACGTTCGGAGCTACGAGGACTTG
 421          430        440        450        460        470        480        490        500        510        520
 421   ACCTCAGGTGCGCCAATCTGAGCCTTTGAACCCGTGGTAGAGAGTCTTGCACTCCCACTTCTCACCAAACCAGATGAGAGATTGCAAGCCTCGATGCTCCTGAAC
 526    TGCTCAGGATGAGAACATCAAAGTCTCTGTGCCTGGTCAGGCCAATGAGCTTCCGCTCCAGACCGTCTTTAGCTCCGACGGTGGAGACACTTACGCTTGGGCTTT
 526     530        540        550        560        570        580        590        600        610        620
 526    ACGAGTCCTACTCTTGTAGTTTCAGAGACACGGACCAGTCCGGTTACTCGAAGGCGAGGTCTGGCAGAAATCGAGGCTGCCACCTCTGTGAATGCGAACCCGAAA
 631    CAAGGCAACTTCTGATGTGGTTAAGGTCACTTTCCATAACCCTGGTGTTCAAGAAGACCGCACGTGTGGACCTCTCTTAGATGTTGTCGCAATCAAGGAGATTCT
 631          640        650        660        670        680        690        700        710        720        730
 631    GTTCCGTTGAAGACTACACCAATTCCAGTGAAAGGTATTGGGACCACAAGTTCTTCTGGCGTGCACACCTGGAGAGAATCTACAACAGCGTTAGTTCCTCTAAGA
 736    TCCTCTCCGGTATACCAGAGGAAACTTGGTGAAAAATGGAGGATTCGAGATCGGTCCACATGTTTTCGCTAATTTCTCAACCGGAATCTTGATTCCAGCAAGAAT
 736     740        750        760        770        780        790        800        810        820        830
 736    AGGAGAGGCCATATGGTCTCCTTTGAACCACTTTTTACCTCCTAAGCTCTAGCCAGGTGTACAAAAGCGATTAAAGAGTTGGCCTTAGAACTAAGGTCGTTCTTA
 841    CCAAGACTTCATCTCTCCTCTTCCGGGATGGATCGTCGAATCCCTTAAACCAGTGAAGTATATCGATAGACGTCATTTCAAAGTTCCTTATGGACAAGGGGCCGT
 841          850        860        870        880        890        900        910        920        930        940
 841    GGTTCTGAAGTAGAGAGGAGAAGGCCCTACCTAGCAGCTTAGGGAATTTGGTCACTTCATATAGCTATCTGCAGTAAAGTTTCAAGGAATACCTGTTCCCCGGCA
 946    CGAGCTAGTTGCCGGAAGAGAAAGCGCAATAGCTCAGATCATCCGAACCATCGCTGGTAAAGCCTATATGCTCTCATTCGCGGTTGGTGATGCACAAAACGGTTG
 946     950        960        970        980        990       1000       1010       1020       1030       1040
 946    GCTCGATCAACGGCCTTCTCTTTCGCGTTATCGAGTCTAGTAGGCTTGGTAGCGACCATTTCGGATATACGAGAGTAAGCGCCAACCACTACGTGTTTTGCCAAC
1051    CCACGGGTCGATGATGGTGGAAGCGTTCGCAGGAAGAGAACCGTTTAAGCTTTCTTTTATGTCAGAAGGTAAAGGAGCGTTCAAAACAGGGCATTTTAGGTTTGT
1051         1060       1070       1080       1090       1100       1110       1120       1130       1140       1150
1051    GGTGCCCAGCTACTACCACCTTCGCAAGCGTCCTTCTCTTGGCAAATTCGAAAGAAAATACAGTCTTCCATTTCCTCGCAAGTTTTGTCCCGTAAAATCCAAACA
1156    TGCTGATTCGGATCGAACGAGGCTAACGTTTTATAGTGCGTTTTATCATACAAAGCTTCATGACTTCGGCCATCTTTGTGGGCCTGTTCTTGATAGCGTTGTTGT
1156    1160       1170       1180       1190       1200       1210       1220       1230       1240       1250
1156    ACGACTAAGCCTAGCTTGCTCCGATTGCAAAATATCACGCAAAATAGTATGTTTCGAAGTACTGAAGCCGGTAGAAACACCCGGACAAGAACTATCGCAACAACA
1261    TACCCTGGCCCGTTAACAATTTGTGGCTTTTAATTCTTATTAGACTGAATATTCGAAACACTATACTCTATTCGTAACGAATTTGTGGTTGCACTACATTTTGTT
1261         1270       1280       1290       1300       1310       1320       1330       1340       1350       1360
1261    ATGGGACCGGGCAATTGTTAAACACCGAAAATTAAGAATAATCTGACTTATAAGCTTTGTGATATGAGATAAGCATTGCTTAAACACCAACGTGATGTAAAACAA
1366    GTTTCTTGTCATTAGTCTTGTCACTCATACCAGTGATGCAGAAAATATTTATAATATGTATGGAAACTTTGTGGAAATTAAACTAAAATTAAAAAGAATGGTGAG
1366    1370       1380       1390       1400       1410       1420       1430       1440       1450       1460
1366   CAAAGAACAGTAATCAGAACAGTGAGTATGGTCACTACGTCTTTTATAAATATTATACATACCTTTGAAACACCTTTAATTTGATTTTAATTTTTCTTACCACTC
1471 ATAGATTTCAATTTAATAATAATTTACATCTTTC
1471         1480       1490       1500
1471 TATCTAAAGTTAAATTATTATTAAATGTAGAAAG
```

Primer:	Sequence:
BDXForward	5'-CTCTCGCTCACTCTTCTCCAA-3'
BDXReverse	5'-CGACAAAGCCTGAGAGTTCCC-3'

Results for linear 1384 residue sequence "AT2G41810.1 | Symbols: no symbol available | no full name available | chr2:17439263-17

```
   1 GCTATATATAGCTTGTAGGAACTTGTGCATATATCACAACCCTCTTATCTTATGTATACTCTTTTATTAGAGACAATTGATAGATTTGTTTTTCAGTGTCACTTG
   1          10        20        30        40        50        60        70        80        90       100
   1 CGATATATATCGAACATCCTTGAACACGTATATAGTGTTGGGAGAATAGAATACATATGAGAAAATAATCTCTGTTAACTATCTAAACAAAAAGTCACAGTGAAC
 106 GCCTAAAAGCTCACAATGTCTCTCGTTCTCTCTCTTTGCTTGGTCTTCCTTTGTTCTCTTGTTCATGGTTCTCATAGCTTACCAGCACAACGGACTCCTCATCTT
 106     110       120       130       140       150       160       170       180       190       200
 106 CGGATTTTCGAGTGTTACAGAGAGCAAGAGAGAGAAACGAACCAGAAGGAAACAAGAGAACAAGTACCAAGAGTATCGAATGGTCGTGTTGCCTGAGGAGTAGAA
 211 GATGGGCTTCTCCCAAATGGGAACTTTGAGCAAATTCCTAACAAATCAAACATGAGAAAGAGGCAAATAATTGGCAAATACTCTCTTCCACATTGGGAAATCTCC
 211          220       230       240       250       260       270       280       290       300       310
 211 CTACCCGAAGAGGGTTTACCCTTGAAACTCGTTTAAGGATTGTTTAGTTTGTACTCTTTCTCCGTTTATTAACCGTTTATGAGAGAAGGTGTAACCCTTTAGAGG
 316 GGCCACGTAGAGCTAGTCTCTGGCGGTCCACAGCCCGGTGGTTTTTACTTCGCAGTGCCACGTGGAGTCCACGCAGCTAGGCTAGGGAACTTAGCATCAATATCT
 316     320       330       340       350       360       370       380       390       400       410
 316 CCGGTGCATCTCGATCAGAGACCGCCAGGTGTCGGGCCACCAAAAATGAAGCGTCACGGTGCACCTCAGGTGCGTCGATCCGATCCCTTGAATCGTAGTTATAGA
 421 CAGTATGTGAAAGTGAAAAGCGGCTTGGTCTATTCTCTAACGTTTGGTGTCACGAGGACTTGTGCTCAAGACGAGAACATCAGAATCTCTGTGCCTGGTCAGACC
 421          430       440       450       460       470       480       490       500       510       520
 421 GTCATACACTTTCACTTTTCGCCGAACCAGATAAGAGATTGCAAACCACAGTGCTCCTGAACACGAGTTCTGCTCTTGTAGTCTTAGAGACACGGACCAGTCTGG
 526 AATGAGCTACCAATCCAAACATTGTTCAGTACCAACGGTGGTGATACGTACGCATGGGCTTTTAAGGCAACGTCTGACTTGGTTAAGGTCACTTTTTATAATCCT
 526     530       540       550       560       570       580       590       600       610       620
 526 TTACTCGATGGTTAGGTTTGTAACAAGTCATGGTTGCCACCACTATGCATGCGTACCCGAAAATTCCGTTGCAGACTGAACCAATTCCAGTGAAAAATATTAGGA
 631 GGTGTTCAAGAAGACCCTACTTGTGGCCCAATCGTTGACGCTGTTGCCATTAAGGAGATTCTTCCTCTCCGGTATACCAAAGGGAACCTTGTGAAAAACGGCGGA
 631          640       650       660       670       680       690       700       710       720       730
 631 CCACAAGTTCTTCTGGGATGAACACCGGGTTAGCAACTGCGACAACGGTAATTCCTCTAAGAAGGAGAGGCCATATGGTTTCCCTTGGAACACTTTTTGCCGCCT
 736 TTTGAGACCGGTCCACACGTATTCAGCAACTTCTCTACCGGAATTTTAATTCCGGCGAAGATACAAGACCTAATCTCACCGCTTCCGGGATGGATCGTCGAATCT
 736     740       750       760       770       780       790       800       810       820       830
 736 AAACTCTGGCCAGGTGTGCATAAGTCGTTGAAGAGATGGCCTTAAAATTAAGGCCGCTTCTATGTTCTGGATTAGAGTGGCGAAGGCCCTACCTAGCAGCTTAGA
 841 CTAAAACCGGTTAAGTACATCGATAACCGCCACTTCAAGGTTCCCTCAGGTCTCGCAGCAATCGAGCTTGTTGCCGGAAGAGAAAGCGCAATCGCTCAGATCATC
 841          850       860       870       880       890       900       910       920       930       940
 841 GATTTTGGCCAATTCATGTAGCTATTGGCGGTGAAGTTCCAAGGGAGTCCAGAGCGTCGTTAGCTCGAACAACGGCCTTCTCTTTCGCGTTAGCGAGTCTAGTAG
 946 CGTACCGTCTCCGGTAAAAACTACATTCTCTCGTTTGTTGTGGGAGACGCACACAATGGTTGTCACGGATCGATGATGGTGGAGGCGTTTGCGGGAATATCGGCG
 946     950       960       970       980       990      1000      1010      1020      1030      1040
 946 GCATGGCAGAGGCCATTTTTGATGTAAGAGAGCAAACAACACCCTCTGCGTGTGTTACCAACAGTGCCTAGCTACTACCACCTCCGCAAACGCCCTTATAGCCGC
1051 TTTAAGGTTACATTCGAATCTAATGATAAAGGAGCGTTCAAAGTGGGGCGTTTCGCGTTTCGTGCCGATTCGAATCGCACAAGGATAACTTTCTATAGTGGGTTT
1051         1060      1070      1080      1090      1100      1110      1120      1130      1140      1150
1051 AAATTCCAATGTAAGCTTAGATTACTATTTCCTCGCAAGTTTCACCCCGCAAAGCGCAAAGCACGGCTAAGCTTAGCGTGTTCCTATTGAAAGATATCACCCAAA
1156 TATCATACCAAGCTTCATGATTTTGGACATCTTTGTGGGCCTGTGCTTGATAATGTTAGTGTTTTTTTGGCCCATTAAATTATTACAAAAGTAAAATAAATATTG
1156    1160      1170      1180      1190      1200      1210      1220      1230      1240      1250
1156 ATAGTATGGTTCGAAGTACTAAAACCTGTAGAAACACCCGGACACGAACTATTACAATCACAAAAAAACCGGGTAATTTAATAATGTTTCATTTTATTTATAAC
1261 TTCGATTTCAATTATATAATTTTCTATACTTCTGTAAAAAGGTCGTTAAGAAACTGATATTCAAAAAATATCGATATTCATATTTCTTGATCCAAAAAATATTGC
1261         1270      1280      1290      1300      1310      1320      1330      1340      1350      1360

1261 AAGCTAAAGTTAATATATTAAAAGATATGAAGACATTTTTCCAGCAATTCTTTGACTATAAGTTTTTTATAGCTATAAGTATAAAGAACTAGGTTTTTTATAACG
1366 ATTGTATTTATATATATCT
1366    1370      1380
1366 TAACATAAATATATATAGA
```

Primer:	Sequence:
BDXForward	5'-CTCTCGCTCACTCTTCTCCAA-3'
BDXReverse	5'-CGACAAAGCCTGAGAGTTCCC-3'

Results for linear 1709 residue sequence "AT3G08030.1 | Symbols: AthA2-1 | | chr3:2563803-2566042 FORWARD LENGTH=1709" starting

```
   1   CTAACTTTAGTTCCTCTCAATTTCTCTTATTACCATACAAAATTTACTAATTACTGTACAAAAATTACCAAATAAAAGGCCACAAATATCTTAGGAAGAAATGAA
   1          10         20         30         40         50         60         70         80         90        100
   1   GATTGAAATCAAGGAGAGTTAAAGAGAATAATGGTATGTTTTAAATGATTAATGACATGTTTTTAATGGTTTATTTTCCGGTGTTTATAGAATCCTTCTTTACTT
 106    GCAAGGCACAAGTGCCAATACGAGATTAGAGAGCATGAATTAAGGAGAAAAGTCGGTTTCGGTTCAGTAAGACAGACAGACAAAATCCATCGTTGGGGTCCAGTC
 106  110  120  130  140  150  160  170  180  190                                                             200
 106   CGTTCCGTGTTCACGGTTATGCTCTAATCTCTCGTACTTAATTCCTCTTTTCAGCCAAAGCCAAGTCATTCTGTCTGTCTGTTTTAGGTAGCAACCCCAGGTCAG
 211    ACATTACCTCTTTCTACGTGATTTCCTCTCTCTCCACTCGCTTTTAATCCTTTCACTTGTATAAAGTCGTAGCATCTCGAGATCGTCTTCCTCTCACAATCTCAA
 211  220  230  240  250  260  270  280  290  300                                                             310
 211   TGTAATGGAGAAAGATGCACTAAAGGAGAGAGAGGTGAGCGAAAATTAGGAAAGTGAACATATTTCAGCATCGTAGAGCTCTAGCAGAAGGAGAGTGTTAGAGTT
 316    ATTCTTCGGACCTTTTCTCTCTCCCCTGAAGCTCCCCTCACTCACTCTTCTCCGAGGAAGAACAACAGAGACAATGGCGGTTCCCAAAGCCATTATTCTACCTAT
 316  320  330  340  350  360  370  380  390  400                                                             410
 316   TAAGAAGCCTGGAAAAGAGAGAGGGGACTTCGAGGGGAGTGAGTGAGAAGAGGCTCCTTCTTGTTGTCTCTGTTACCGCCAAGGGTTTCGGTAATAAGATGGATA
 421    CTTGCTACTCATCTGTGGTGCTGCTCTCGGAGCTCCTGCTTCTGAAGGTTATCTTCGTAATGGAAACTTCGAAGAGTCACCAAAGAAAACCGACATGAAGAAAAC
 421  430  440  450  460  470  480  490  500  510                                                             520
 421   GAACGATGAGTAGACACCACGACGAGAGCCTCGAGGACGAAGACTTCCAATAGAAGCATTACCTTTGAAGCTTCTCAGTGGTTTCTTTTGGCTGTACTTCTTTTG
 526    AGTTCTACTAGGCAAAAACGCCTTGCCCGAATGGGAAACCACCGGTTTCGTCGAGTACATCGCCGGCGGTCCTCAGCCAGGAGGCATGTACTTCCCAGTGGCTCA
 526  530  540  550  560  570  580  590  600  610                                                             620
 526    TCAAGATGATCCGTTTTTGCGGAACGGGCTTACCCTTTGGTGGCCAAAGCAGCTCATGTAGCGGCCGCCAGGAGTCGGTCCTCCGTACATGAAGGGTCACCGAGT
 631    TGGAGTCCACGCCGTGAGGCTCGGAAACGAAGCTACAATCTCTCAGAAGTTAGAAGTGAAGCCAGGTTCTCTCTACGCACTCACGTTTGGTGCGTCGAGAACTTG
 631  640  650  660  670  680  690  700  710  720                                                             730
 631    ACCTCAGGTGCGGCACTCCGAGCCTTTGCTTCGATGTTAGAGAGTCTTCAATCTTCACTTCGGTCCAAGAGAGATGCGTGAGTGCAAACCACGCAGCTCTTGAAC
 736    TGCACAAGACGAAGTTCTTAGAGTCTCTGTACCTTCTCAGTCCGGTGACTTGCCACTTCAAACACTTTACAACAGTTTCGGAGGTGACGTGTACGCTTGGGCCTT
 736     740        750        760        770        780        790        800        810        820        830
 736    ACGTGTTCTGCTTCAAGAATCTCAGAGACATGGAAGAGTCAGGCCACTGAACGGTGAAGTTTGTGAAATGTTGTCAAAGCCTCCACTGCACATGCGAACCCGGAA
 841    CGTCGCCAAGACTTCTCAAGTTACTGTGACTTTCCACAATCCTGGAGTTCAAGAAGATCCTGCTTGTGGTCCTTTGTTGGACGCTGTCGCCATTAAAGAGCTTGT
 841          850        860        870        880        890        900        910        920        930        940
 841    GCAGCGGTTCTGAAGAGTTCAATGACACTGAAAGGTGTTAGGACCTCAAGTTCTTCTAGGACGAACACCAGGAAACAACCTGCGACAGCGGTAATTTCTCGAACA
 946    TCATCCAATCTACACCAGAGGGAACTTGGTGAAGAATGGAGGGTTCGAAGAAGGTCCTCACCGTCTAGTGAACTCCACACAAGGAGTCCTACTCCCACCTAAACA
 946     950        960        970        980        990       1000       1010       1020       1030       1040
 946    AGTAGGTTAGATGTGGTCTCCCTTGAACCACTTCTTACCTCCCAAGCTTCTTCCAGGAGTGGCAGATCACTTGAGGTGTGTTCCTCAGGATGAGGGTGGATTTGT
1051    AGAAGATCTCACATCACCTTTACCTGGTTGGATCATAGAGTCACTCAAGGCAGTGAAATTCATAGACTCTAAGTACTTTAATGTCCCCTTTGGACATGCTGCGAT
1051         1060       1070       1080       1090       1100       1110       1120       1130       1140       1150
1051    TCTTCTAGAGTGTAGTGGAAATGGACCAACCTAGTATCTCAGTGAGTTCCGTCACTTTAAGTATCTGAGATTCATGAAATTACAGGGGAAACCTGTACGACGCTA
1156    CGAGCTAGTTGCAGGCAAAGAAAGTGCCATTGCACAAGTCATTAGAACCTCTCCTGGTCAAACTTACACCCTCTCCTTCGTCGTTGGAGATGCTAAGAATGACTG
1156    1160       1170       1180       1190       1200       1210       1220       1230       1240       1250
1156    GCTCGATCAACGTCCGTTTCTTTCACGGTAACGTGTTCAGTAATCTTGGAGAGGACCAGTTTGAATGTGGGAGAGGAAGCAGCAACCTCTACGATTCTTACTGAC
1261    CCATGGTTCCATGATGGTTGAAGCTTTTGCAGCCAGAGATACACTCAAAGTACCACACACTTCCGTTGGTGGAGGTCATGTCAAGACCGCGAGTTTCAAGTTCAA
1261         1270       1280       1290       1300       1310       1320       1330       1340       1350       1360
1261    GGTACCAAGGTACTACCAACTTCGAAAACGTCGGTCTCTATGTGAGTTTCATGGTGTGTGAAGGCAACCACCTCCAGTACAGTTCTGGCGCTCAAAGTTCAAGTT
1366    GGCGGTTGAGGCAAGAACTAGAATTACTTTCTTCAGTGGTTTTTACCATACCAAGAAGACTGATACTGTATCTTTATGTGGTCCTGTCATTGACGAGATTGTGGT
1366    1370       1380       1390       1400       1410       1420       1430       1440       1450       1460
1366    CCGCCAACTCCGTTCTTGATCTTAATGAAAGAAGTCACCAAAAATGGTATGGTTCTTCTGACTATGACATAGAAATACACCAGGACAGTAACTGCTCTAACACCA
1471    TTCTCATGTCGCTTAGATTAAGTTTACTCTTTATGACAACTGCACTTGTGTTCTTAGACTGGTCATGGACTTGGTGCCTTGCTTTGTATGTCTTGGCTCCTTTTG
1471         1480       1490       1500       1510       1520       1530       1540       1550       1560       1570
1471    AAGAGTACAGCGAATCTAATTCAAATGAGAAATACTGTTGACGTGAACACAAGAATCTGACCAGTACCTGAACCACGGAACGAAACATACAGAACCGAGGAAAAC
1576    GAATTGAGTTATTGTCATCAAGTTAAAATGAATGATATATTATTGTAATATGAAATGTTTTGAGTTAAACGAATACCAAGAAGTATAATCTAGGACATTCCAAAA
1576    1580       1590       1600       1610       1620       1630       1640       1650       1660       1670
1576   CTTAACTCAATAACAGTAGTTCAATTTTACTTACTATATAATAACATTATACTTTACAAAACTCAATTTGCTTATGGTTCTTCATATTAGATCCTGTAAGGTTTT
1681 AGTGCAAGGAAATTAATACCTATAAAGTA
1681         1690       1700
1681 TCACGTTCCTTTAATTATGGATATTTCAT
```

Primer:	Sequence:
BDXForward	5'-CTCTCGCTCACTCTTCTCCAA-3'
BDXReverse	5'-CGACAAAGCCTGAGAGTTCCC-3'

Results for linear 1508 residue sequence "AT4G32460.1 | Symbols: AthA1-1, BDX, BIIDXI | | chr4:15662799-15665032 REVERSE LENGTH=

```
                                                                                           >>>BDXForward>>> 81 to 101
   1    AAGGGGCCTTTCTTGCACAATTTTGCATATAAGAGCTCTCTCTCCTCCTCGTTCTATTCCACTCCCACTAAACATTCCTTCTCTCGCTCACTCTTCTCCAATCCT
   1            10        20        30        40        50        60        70        80        90        100
   1    TTCCCCGGAAAGAACGTGTTAAAACGTATATTCTCGAGAGAGAGGAGGAGCAAGATAAGGTGAGGGTGATTTGTAAGGAAGAGAGCGAGTGAGAAGAGGTTAGGA
 106     TATTTTATTTTTTGAAAGTTTAAAATTTTATACAACATATCAATTTGGGGTAGAAAAATTCGAAAGAAATGAAAGAGATGGGAGTGATAGTGCTTCTTCTCCTTC
 106      110       120       130       140       150       160       170       180       190       200
 106    ATAAAATAAAAAACTTTCAAATTTTAAAATATGTTGTATAGTTAAACCCCATCTTTTTAAGCTTTCTTTACTTTCTCTACCCTCACTATCACGAAGAAGAGGAAG
 211     ACTCGTTCTTCTACGTTGCCTTTTGCTTCAATGATGGACTACTACCAAACGGTGACTTCGAACTCGGTCCACGACATTCGGACATGAAAGGAACACAAGTTATCA
 211            220       230       240       250       260       270       280       290       300       310
 211    TGAGCAAGAAGATGCAACGGAAAACGAAGTTACTACCTGATGATGGTTTGCCACTGAAGCTTGAGCCAGGTGCTGTAAGCCTGTACTTTCCTTGTGTTCAATAGT
                          <<<BDXReverse<<< 337 to 357
 316     ACATAACAGCAATCCCAAACTGGGAACTCTCAGGCTTTGTCGAGTACATTCCCTCAGGACACAAACAAGGCGACATGATCCTTGTCGTGCCTAAAGGCGCATTCG
 316      320       330       340       350       360       370       380       390       400       410
 316     TGTATTGTCGTTAGGGTTTGACCCTTGAGAGTCCGAAACAGCTCATGTAAGGGAGTCCTGTGTTTGTTCCGCTGTACTAGGAACAGCACGGATTTCCGCGTAAGC
 421     CAGTACGTCTAGGCAACGAAGCCTCAATCAAACAAAAAATCAGCGTTAAGAAAGGGTCGTACTATTCGATAACGTTCAGTGCTGCTCGAACATGCGCACAAGACG
 421            430       440       450       460       470       480       490       500       510       520
 421 GTCATGCAGATCCGTTGCTTCGGAGTTAGTTTGTTTTTTAGTCGCAATTCTTTCCCAGCATGATAAGCTATTGCAAGTCACGACGAGCTTGTACGCGTGTTCTGC
 526 AGCGGTTAAACGTTTCCGTGGCTCCTCACCATGCAGTGATGCCGATACAAACAGTGTATAGTAGCTCAGGTTGGGATTTGTATTCGTGGGCTTTTAAGGCCCAAA
 526     530       540       550       560       570       580       590       600       610       620
 526 TCGCCAATTTGCAAAGGCACCGAGGAGTGGTACGTCACTACGGCTATGTTTGTCACATATCATCGAGTCCAACCCTAAACATAAGCACCCGAAAATTCCGGGTTT
 631 GTGACTATGCAGATATAGTGATACATAATCCAGGTGTTGAGGAAGATCCTGCTTGTGGACCTCTCATTGATGGTGTTGCTATGCGAGCCCTTTTCCCTCCTCGTC
 631          640       650       660       670       680       690       700       710       720       730
 631 CACTGATACGTCTATATCACTATGTATTAGGTCCACAACTCCTTCTAGGACGAACACCTGGAGAGTAACTACCACAACGATACGCTCGGGAAAAGGGAGGAGCAG
 736 CCACCAATAAGAACATTCTAAAGAACGGAGGATTCGAAGAAGGTCCTTGGGTTTTACCAAACATATCATCTGGTGTTTTGATTCCACCAAACTCCATCGACGATC
 736     740       750       760       770       780       790       800       810       820       830
 736 GGTGGTTATTCTTGTAAGATTTCTTGCCTCCTAAGCTTCTTCCAGGAACCCAAAATGGTTTGTATAGTAGACCACAAAACTAAGGTGGTTTGAGGTAGCTGCTAG
 841 ACTCTCCGTTACCTGGTTGGATGGTCGAGTCTCTTAAAGCTGTCAAATACATAGATTCCGATCATTTCTCCGTTCCTCAAGGCCGTCGCGCCGTCGAACTCGTCG
 841          850       860       870       880       890       900       910       920       930       940
 841 TGAGAGGCAATGGACCAACCTACCAGCTCAGAGAATTTCGACAGTTTATGTATCTAAGGCTAGTAAAGAGGCAAGGAGTTCCGGCAGCGCGGCAGCTTGAGCAGC
 946 CCGGGAAAGAAAGCGCCGTCGCACAAGTTGTCCGCACTATCCCTGGAAAAACCTACGTCCTATCCTTCTCTGTCGGAGATGCTAGCAACGCTTGCGCCGGATCAA
 946     950       960       970       980       990       1000      1010      1020      1030      1040
 946 GGCCCTTTCTTTCGCGGCAGCGTGTTCAACAGGCGTGATAGGGACCTTTTTGGATGCAGGATAGGAAGAGACAGCCTCTACGATCGTTGCGAACGCGGCCTAGTT
1051 TGATCGTCGAAGCTTTCGCCGGAAAAGACACGATCAAGGTCCCGTATGAATCGAAAGGGAAAGGAGGATTCAAGCGATCGTCATTGAGATTCGTCGCTGTCTCGA
1051          1060      1070      1080      1090      1100      1110      1120      1130      1140      1150
1051 ACTAGCAGCTTCGAAAGCGGCCTTTTCTGTGCTAGTTCCAGGGCATACTTAGCTTTCCCTTTCCTCCTAAGTTCGCTAGCAGTAACTCTAAGCAGCGACAGAGCT
1156 GTCGGACTAGAGTTATGTTCTACAGTACGTTTTACGCGATGAGAAACGACGATTTCTCGAGCTTATGTGGACCGGTGATCGACGACGTTAAGCTTCTCAGTGCTC
1156     1160      1170      1180      1190      1200      1210      1220      1230      1240      1250
1156 CAGCCTGATCTCAATACAAGATGTCATGCAAAATGCGCTACTCTTTGCTGCTAAAGAGCTCGAATACACCTGGCCACTAGCTGCTGCAATTCGAAGAGTCACGAG
1261 GGAGGCCGTGAGCTTGCGGCGACGAGTTGATTCACGGGACAATGAATGATGACAGTCACTGTGGGTTTCTCGCGTCTAGTGAGAAATTGGGCTTTTAGGCCCAGT
1261          1270      1280      1290      1300      1310      1320      1330      1340      1350      1360
1261 CCTCCGGCACTCGAACGCCGCTGCTCAACTAAGTGCCCTGTTACTTACTACTGTCAGTGACACCCAAAGAGCGCAGATCACTCTTTAACCCGAAAATCCGGGTCA
1366 GGCCCACTGTTTTTGTTGTTGTTTTAAAGCTTAATGTTATTTGACAAAGAAAAAAGAAATTACTCTGGTCAATCATATCGAACCGTGAAATTTTATGATCTTGTG
1366     1370      1380      1390      1400      1410      1420      1430      1440      1450      1460
1366 CCGGGTGACAAAAACAACAACAAAATTTCGAATTACAATAAACTGTTTCTTTTTTCTTTAATGAGACCAGTTAGTATAGCTTGGCACTTTAAAATACTAGAACAC
1471 ATTTAAGAATGTTTCTTCCTCACTGAGTGGACAAATAC
1471          1480      1490      1500
1471 TAAATTCTTACAAAGAAGGAGTGACTCACCTGTTTATG
```

Primer:	Sequence:
BDXForward	5'-CTCTCGCTCACTCTTCTCCAA-3'
BDXReverse	5'-CGACAAAGCCTGAGAGTTCCC-3'

Results for linear 1629 residue sequence "AT5G11420.1 | Symbols: no symbol available | no full name available | chr5:3644547-364

```
   1   TTTTCCAAGTTAGACTTCCATATAAGCTATTGCTGGCTCCTCTCCCAATTTCCCATAATTTACTAAATATCACTTTCTAGTCTCTTCTCTTTACTTTGGTCGTCC
   1          10        20        30        40        50        60        70        80        90       100
   1   AAAAGGTTCAATCTGAAGGTATATTCGATAACGACCGAGGAGAGGGTTAAAGGGTATTAAATGATTTATAGTGAAAGATCAGAGAAGAGAAATGAAACCAGCAGG
 106    ACAATGAAAGGAGGCAGCCTCTCGTTTCTCTTCGTTCTCCTAATCGCCACCATCACTTCCGTCATTTGCTTCAGTGACGGGATGTTACCAAACGGCGACTTTGAA
 106  110  120  130  140  150  160  170  180  190                                                         200
 106   TGTTACTTTCCTCCGTCGGAGAGCAAAGAGAAGCAAGAGGATTAGCGGTGGTAGTGAAGGCAGTAAACGAAGTCACTGCCCTACAATGGTTTGCCGCTGAAACTT
 211    CTAGGACCAAAACCATCGGACATGAAAGGAACGCAAGTAATAAACAAGAAGGCGATTCCTAGCTGGGAGCTTTCAGGCTTCGTCGAATACATAAAGTCCGGTCAA
 211  220  230  240  250  260  270  280  290  300                                                         310
 211   GATCCTGGTTTTGGTAGCCTGTACTTTCCTTGCGTTCATTATTTGTTCTTCCGCTAAGGATCGACCCTCGAAAGTCCGAAGCAGCTTATGTATTTCAGGCCAGTT
 316    AAACAAGGAGACATGCTTCTCGTAGTCCCGGCCGGAAAGTTCGCAATCCGGCTAGGCAACGAGGCATCGATCAAACAAAGACTTAACGTTACAAAAGGAATGTAT
 316  320  330  340  350  360  370  380  390  400                                                         410
 316   TTTGTTCCTCTGTACGAAGAGCATCAGGGCCGGCCTTTCAAGCGTTAGGCCGATCCGTTGCTCCGTAGCTAGTTTGTTTCTGAATTGCAATGTTTTCCTTACATA
 421    TACTCACTGACGTTCAGTGCCGCAAGGACATGTGCCCAAGACGAACGGCTCAACATATCGGTGGCACCTGACTCAGGCGTTATTCCTATACAGACGGTGTACAGT
 421  430  440  450  460  470  480  490  500  510                                                         520
 421   ATGAGTGACTGCAAGTCACGGCGTTCCTGTACACGGGTTCTGCTTGCCGAGTTGTATAGCCACCGTGGACTGAGTCCGCAATAAGGATATGTCTGCCACATGTCA
 526    AGCAGTGGATGGGACCTTTACGCATGGGCGTTCCAAGCCGAGAGTAACGTGGCAGAGATCGTGATTCATAATCCTGGTGAGGAGGAAGATCCTGCTTGTGGACCA
 526  530  540  550  560  570  580  590  600  610                                                         620
 526    TCGTCACCTACCCTGGAAATGCGTACCCGCAAGGTTCGGCTCTCATTGCACCGTCTCTAGCACTAAGTATTAGGACCACTCCTCCTTCTAGGACGAACACCTGGT
 631    CTCATTGATGGTGTGGCAATCAAAGCTCTATACCCTCCTCGGCCCACCAATAAGAATATATTGAAGAACGGAGGATTTGAAGAAGGTCCCTACGTACTCCCAAAC
 631  640  650  660  670  680  690  700  710  720                                                         730
 631    GAGTAACTACCACACCGTTAGTTTCGAGATATGGGAGGAGCCGGGTGGTTATTCTTATATAACTTCTTGCCTCCTAAACTTCTTCCAGGGATGCATGAGGGTTTG
 736    GCAACAACCGGCGTTCTGGTTCCTCCCTTTATAGAAGATGACCACTCTCCTTTACCCGCGTGGATGGTCGAATCACTCAAAGCCATCAAATACGTTGATGTCGAG
 736     740       750       760       770       780       790       800       810       820       830
 736    CGTTGTTGGCCGCAAGACCAAGGAGGGAAATATCTTCTACTGGTGAGAGGAAATGGGCGCACCTACCAGCTTAGTGAGTTTCGGTAGTTTATGCAACTACAGCTC
 841    CATTTCTCGGTCCCACAAGGCCGTCGAGCCGTGGAGCTAGTGGCAGGCAAAGAAAGCGCAATCGCTCAGGTAGCTAGGACCGTTGTGGGAAAAACTTACGTGCTT
 841          850       860       870       880       890       900       910       920       930       940
 841    GTAAAGAGCCAGGGTGTTCCGGCAGCTCGGCACCTCGATCACCGTCCGTTTCTTTCGCGTTAGCGAGTCCATCGATCCTGGCAACACCCTTTTTGAATGCACGAA
 946    TCGTTTGCGGTTGGAGATGCTAACAATGCTTGCCAAGGATCGATGGTGGTCGAGGCATTTGCGGGAAAAGACACTCTAAAGGTACCTTATGAGTCTCGAGGCAAA
 946     950       960       970       980       990      1000      1010      1020      1030      1040
 946    AGCAAACGCCAACCTCTACGATTGTTACGAACGGTTCCTAGCTACCACCAGCTCCGTAAACGCCCTTTTCTGTGAGATTTCCATGGAATACTCAGAGCTCCGTTT
1051    GGAGGGTTCAAACGCGCTTCTCTACGGTTTGTGGCGGTTTCGACCCGCACAAGAGTTATGTTTTACAGCACATTTTACTCGATGAGAAGCGATGATTTCTCATCA
1051         1060      1070      1080      1090      1100      1110      1120      1130      1140      1150
1051    CCTCCCAAGTTTGCGCGAAGAGATGCCAAACACCGCCAAAGCTGGGCGTGTTCTCAATACAAAATGTCGTGTAAAATGAGCTACTCTTCGCTACTAAAGAGTAGT
1156    CTGTGTGGGCCCGTGATCGATGATGTTAAGCTCCTCAGTGCTCGTAAGCCGTAAGATGGCCATTGTTTTATTTCTAAGGTTCTTCGTATAAGCCAGTGTGGTTTT
1156     1160      1170      1180      1190      1200      1210      1220      1230      1240      1250
1156    GACACACCCGGGCACTAGCTACTACAATTCGAGGAGTCACGAGCATTCGGCATTCTACCGGTAACAAAATAAAGATTCCAAGAAGCATATTCGGTCACACCAAAA
1261    TTATATAAATATGATTTTCTTAATCTCTAATGCTTTTGAAATGAGATGAGTAATACTGAAAGGGTGTTTGTGGTGTAGGGTTTTATATAAGGCAAAAAGGTCTTG
1261         1270      1280      1290      1300      1310      1320      1330      1340      1350      1360
1261    AATATATTTATACTAAAAGAATTAGAGATTACGAAAACTTTACTCTACTCATTATGACTTTCCCACAAACACCACATCCCAAAATATATTCCGTTTTTCCAGAAC
1366    CTTATATATATTTTCTTGTATGAGAAGGCTAAAGAAATGTTGTCTGTTTTTACTAAATTGGGTTCTTTCAAGGAGATCGGGTGATTGTGAGAATAAAAATGTAAT
1366     1370      1380      1390      1400      1410      1420      1430      1440      1450      1460
1366    GAATATATATAAAAGAACATACTCTTCCGATTTCTTTACAACAGACAAAAATGATTTAACCCAAGAAAGTTCCTCTAGCCCACTAACACTCTTATTTTTACATTA
1471    ACAGATATATATGGTAGAGAGGAATGAGGATAATAATTTAACTTTAGCTTCTTGTGTACTACCAATCCGTGTTAGGATCAAGCTTTTCTAATCAGTTTCAAACTG
1471         1480      1490      1500      1510      1520      1530      1540      1550      1560      1570
1471   TGTCTATATATATACCATCTCTCCTTACTCCTATTATTAAATTGAAATCGAAGAACACATGATGGTTAGGCACAATCCTAGTTCGAAAAGATTAGTCAAAGTTTGAC
1576  AACTGCCTTGAAATCCATGTTAGTAGCTGAAGCAATGGCTCAAAATTTTAAAAC
1576     1580      1590      1600      1610      1620
1576  TTGACGGAACTTTAGGTACAATCATCGACTTCGTTACCGAGTTTTAAAATTTTG
```

Primer:	Sequence:
BDXForward	5'-CTCTCGCTCACTCTTCTCCAA-3'
BDXReverse	5'-CGACAAAGCCTGAGAGTTCCC-3'

Results for linear 1453 residue sequence "AT5G14150.1 | Symbols: AthD-1 | | chr5:4565135-4566988 REVERSE LENGTH=1453" starting "

```
   1   AAATAGAACATTGTTCACACTCTCAATTCACCAACCTTTTGTTTCCTGAAATGTACAAACACTAGAACCAGATCCAAAAAGACACAGTTCTACTATATGGATGAT
   1          10         20         30         40         50         60         70         80         90        100
   1   TTTATCTTGTAACAAGTGTGAGAGTTAAGTGGTTGGAAAACAAAGGACTTTACATGTTTGTGATCTTGGTCTAGGTTTTTCTGTGTCAAGATGATATACCTACTA
 106   GGCCCTGGCTTCTTCAAACCATCTGTGTGTTTAGTCTTCTCATTTGTGCACAACTCAAAGACAAGTCCACTAAGTTTGAAGCAAAATGGCGATATGGTTTCAGAG
 106  110  120  130  140  150  160  170  180  190                                                         200
 106   CCGGGACCGAAGAAGTTTGGTAGACACACAAATCAGAAGAGTAAACACGTGTTGAGTTTCTGTTCAGGTGATTCAAACTTCGTTTTACCGCTATACCAAAGTCTC
 211   AATTTTCCTGTTGCTTCTCGTTTCCTGTTGCGCCTCTTCAGATTTTTTGGAAAATCCAGACTTCGAATCTCCACCGTTAAACTTACCTACAAACTCAAATGCTAG
 211  220  230  240  250  260  270  280  290  300                                                         310
 211   TTAAAAGGACAACGAAGAGCAAAGGACAACGCGGAGAAGTCTAAAAAACCTTTTAGGTCTGAAGCTTAGAGGTGGCAATTTGAATGGATGTTTGAGTTTACGATC
 316   CTCTGTATCGCTGGATCAGAACAGTACACTCCCGGGATGGACATTCCAAGGGACAGTGCTTTATGTTGAACTGCCTGATACCGGACACGCGGTTCAGCTCGGTGA
 316  320  330  340  350  360  370  380  390  400                                                         410
 316   GAGACATAGCGACCTAGTCTTGTCATGTGAGGGCCCTACCTGTAAGGTTCCCTGTCACGAAATACAACTTGACGGACTATGGCCTGTGCGCCAAGTCGAGCCACT
 421   AGATGGCAAGATCAATCAAACATTCATTGCCAAAGGTGATGAATTGAACTACATCCTCACATTCGCACTGATCCACGCAGGCCAGAACTGTACAAGCTCTGCTGG
 421  430  440  450  460  470  480  490  500  510                                                         520
 421   TCTACCGTTCTAGTTAGTTTGTAAGTAACGGTTTCCACTACTTAACTTGATGTAGGAGTGTAAGCGTGACTAGGTGCGTCCGGTCTTGACATGTTCGAGACGACC
 526   TCTCAGTGTCTCGGGGCCAGACAGCAATGCGGTCTTTTCTTACAGACAAAACTACAGTAAGGTTTCATGGCAGAGCTACAGTCATAACTTGGGTAGTTGGGGGAA
 526  530  540  550  560  570  580  590  600  610                                                         620
 526   AGAGTCACAGAGCCCCGGTCTGTCGTTACGCCAGAAAAGAATGTCTGTTTTGATGTCATTCCAAAGTACCGTCTCGATGTCAGTATTGAACCCATCAACCCCCTT
 631   TGGTGAGCCTATTAACTTAGTTCTTGAAAGTCAGGCAATAGATTCTGATTCTGATACAAACTCCACATGTTGGCCTATCATTGACACATTGCTTATCAAGACTGT
 631  640  650  660  670  680  690  700  710  720                                                         730
 631   ACCACTCGGATAATTGAATCAAGAACTTTCAGTCCGTTATCTAAGACTAAGACTATGTTTGAGGTGTACAACCGGATAGTAACTGTGTAACGAATAGTTCTGACA
 736   TGGTGTAACATTGGTCCAAGACAGTGGTAACCTCTTAATCAATGGTGGATTTGAGTCTGGACCTGGTTTCTTGCCCAACTCAACCGATGGAGTTCTGATTGACGC
 736    740        750        760        770        780        790        800        810        820        830
 736   ACCACATTGTAACCAGGTTCTGTCACCATTGGAGAATTAGTTACCACCTAAACTCAGACCTGGACCAAAGAACGGGTTGAGTTGGCTACCTCAAGACTAACTGCG
 841   GGTTCCAAGCCTGATTCAATCACCATTAAGGCAGTGGTCTGTCATAGGAACAGTCAGATACATAGACTCGGAGCACTTCCATGTCCCAGAAGGCAAAGCTGCAAT
 841          850        860        870        880        890        900        910        920        930        940
 841   CCAAGGTTCGGACTAAGTTAGTGGTAATTCCGTCACCAGACAGTATCCTTGTCAGTCTATGTATCTGAGCCTCGTGAAGGTACAGGGTCTTCCGTTTCGACGTTA
 946   AGAAATTCTGTCTAACACAGCTCCATCTGGCATACAGACAGCAACAAAAGGCACAAGTGAAGGTTCAAGATACAACCTCACATTTACCTTAGGCGATGCTAACGA
 946    950        960        970        980        990       1000       1010       1020       1030       1040
 946   TCTTTAAGACAGATTGTGTCGAGGTAGACCGTATGTCTGTCGTTGTTTTCCGTGTTCACTTCCAAGTTCTATGTTGGAGTGTAAATGGAATCCGCTACGATTGCT
1051   TGCGTGCAGAGGACATTTCGTGGTGGGTGCTCAAGCTGGCTCCGTAACTCAAAACTTCACATTGGAGAGTAATGGGACTGGCTCTGGTGAGAAGTTTGGGTTAGT
1051         1060       1070       1080       1090       1100       1110       1120       1130       1140       1150
1051   ACGCACGTCTCCTGTAAAGCACCACCCACGAGTTCGACCGAGGCATTGAGTTTTGAAGTGTAACCTCTCATTACCCTGACCGAGACCACTCTTCAAACCCAATCA
1156   GTTTGAAGCAGACAAAGATGCAGCACAGATAAGTTTCACCAGCTACTCAGTTACAATGACAAAAGAGAATGTTGTTTGTGGTCCTGTGATTGATGAAGTGATGGT
1156   1160       1170       1180       1190       1200       1210       1220       1230       1240       1250
1156   CAAACTTCGTCTGTTTCTACGTCGTGTCTATTCAAAGTGGTCGATGAGTCAATGTTACTGTTTTCTCTTACAACAAACACCAGGACACTAACTACTTCACTACCA
1261   ACATCCTCTTGGTGGAACAGCCTCAGTAAAACCCACTTGGCTGCTACTTATTTTCGCTTTGTTGTATGTTGCAGTTCTCTGATTGAAACTGTTTGGGAGAGAGGT
1261         1270       1280       1290       1300       1310       1320       1330       1340       1350       1360
1261   TGTAGGAGAACCACCTTGTCGGAGTCATTTTGGGTGAACCGACGATGAATAAAAGCGAAACAACATACAACGTCAAGAGACTAACTTTGACAAACCCTCTCTCCA
1366   CATAAAGAAGATATTTTTGCAAGGAGATGTGATAATCATTCTAGCTTCATGATTTATATATGAACACATTCGTTACACATGAATCTTT
1366   1370       1380       1390       1400       1410       1420       1430       1440       1450
1366   GTATTTCTTCTATAAAAACGTTCCTCTACACTATTAGTAAGATCGAAGTACTAAATATATACTTGTGTAAGCAATGTGTACTTAGAAA
```

Primer:	Sequence:
BDXForward	5'-CTCTCGCTCACTCTTCTCCAA-3'
BDXReverse	5'-CGACAAAGCCTGAGAGTTCCC-3'

Results for linear 1599 residue sequence "AT5G25460.1 | Symbols: DGR2 | DUF642 L-GalL responsive gene 2 | chr5:8863224-8865677 F

```
   1   CCCCAAATGGTCAATAAGGTTCCAAATCTCTGAAAAGGAATGAGGTCACAACCCCACGTGGGATACCAACTAGCCACAGTGGCACACACTCGTAATATATAGAAA
   1          10        20        30        40        50        60        70        80        90       100
   1   GGGGTTTACCAGTTATTCCAAGGTTTAGAGACTTTTCCTTACTCCAGTGTTGGGGTGCACCCTATGGTTGATCGGTGTCACCGTGTGTGAGCATTATATATCTTT
 106    CCTCCAAGGTCATTTTCATAGTTCTATCCCCTTATAAGCAATAGCCACAACTCTCTTTGCTAATTACATTTTACACTCATCTCGTTCTCTCTTCCGTCAATATGG
 106     110       120       130       140       150       160       170       180       190       200
 106   GGAGGTTCCAGTAAAAGTATCAAGATAGGGGAATATTCGTTATCGGTGTTGAGAGAAACGATTAATGTAAAATGTGAGTAGAGCAAGAGAGAAGGCAGTTATACC
 211    AAGGCGTCACCGTCGTGTCTTTCTTCCTTCTTTTCATCGCCACCGCCATGGCCGCCAAGTCCACCGTCTCCTTCCGTGACGGCATGTTACCAAACGGAGACTTCG
 211          220       230       240       250       260       270       280       290       300       310
 211   TTCCGCAGTGGCAGCACAGAAAGAAGGAAGAAAAGTAGCGGTGGCGGTACCGGCGGTTCAGGTGGCAGAGGAAGGCACTGCCGTACAATGGTTTGCCTCTGAAGC
 316    AGCTAGGACCAAAACCATCAGACATGAAAGGAACAGAAATACTAAACAAACTAGCAATACCAAACTGGGAAGTCACAGGATTCGTCGAATACATTAAATCAGGAC
 316     320       330       340       350       360       370       380       390       400       410
 316   TCGATCCTGGTTTTGGTAGTCTGTACTTTCCTTGTCTTTATGATTTGTTTGATCGTTATGGTTTGACCCTTCAGTGTCCTAAGCAGCTTATGTAATTTAGTCCTG
 421    ATAAACAAGGAGACATGCTTCTCGTTGTTCCCGCCGGTAAATTCGCTGTAAGACTTGGGAACGAAGCATCGATCAAACAAAGACTTAAAGTGGTTAAAGGAATGT
 421          430       440       450       460       470       480       490       500       510       520
 421   TATTTGTTCCTCTGTACGAAGAGCAACAAGGGCGGCCATTTAAGCGACATTCTGAACCCTTGCTTCGTAGCTAGTTTGTTTCTGAATTTCACCAATTTCCTTACA
 526    ATTACTCACTCACTTTTAGTGCTGCTAGAACTTGTGCACAAGACGAGAGACTTAACATATCTGTAGCACCTGACTCCGGTGTGATTCCGATTCAGACGGTTTATA
 526     530       540       550       560       570       580       590       600       610       620
 526    TAATGAGTGAGTGAAAATCACGACGATCTTGAACACGTGTTCTGCTCTCTGAATTGTATAGACATCGTGGACTGAGGCCACACTAAGGCTAAGTCTGCCAAATAT
 631    GTAGTAGTGGTTGGGATTTATATGCTTGGGCGTTTCAAGCTGAGAGTGATGTTGCTGAAGTTGTGATTCATAATCCTGGTGTTGAGGAAGATCCAGCTTGTGGTC
 631          640       650       660       670       680       690       700       710       720       730
 631    CATCATCACCAACCCTAAATATACGAACCCGCAAAGTTCGACTCTCACTACAACGACTTCAACACTAAGTATTAGGACCACAACTCCTTCTAGGTCGAACACCAG
 736    CACTTATTGATGGTGTTGCTATGAGATCTCTTTACCCTCCTAGACCAACTAATAAGAACATTTTGAAAAACGGAGGATTTGAAGAAGGTCCATTAGTATTACCCG
 736     740       750       760       770       780       790       800       810       820       830
 736    GTGAATAACTACCACAACGATACTCTAGAGAAATGGGAGGATCTGGTTGATTATTCTTGTAAAACTTTTTGCCTCCTAAACTTCTTCCAGGTAATCATAATGGGC
 841    GCTCGACAACTGGAGTTTTGATCCCACCGTTTATAGAAGACGACCACTCTCCTTTACCTGGATGGATGGTGGAGTCTCTCAAAGCTGTCAAGTACGTAGACGTTG
 841          850       860       870       880       890       900       910       920       930       940
 841    CGAGCTGTTGACCTCAAAACTAGGGTGGCAAATATCTTCTGCTGGTGAGAGGAAATGGACCTACCTACCACCTCAGAGAGTTTCGACAGTTCATGCATCTGCAAC
 946    AACATTTCTCAGTCCCACAGGGTCGCAGAGCTATTGAGCTTGTAGCGGGTAAAGAGAGTGCCATCGCTCAAGTGGTTCGGACTGTCATTGGGAAGACTTACGTGC
 946     950       960       970       980       990      1000      1010      1020      1030      1040
 946   TTGTAAAGAGTCAGGGTGTCCCAGCGTCTCGATAACTCGAACATCGCCCATTTCTCTCACGGTAGCGAGTTCACCAAGCCTGACAGTAACCCTTCTGAATGCACG
1051   TGTCTTTTGCGGTTGGAGACGCCAACAATGCTTGCAAAGGATCAATGGTGGTTGAGGCTTTTGCAGGAAAAGATACACTTAAGGTCCCTTACGAGTCGAAAGGCA
1051      1060     1070     1080     1090     1100     1110     1120     1130     1140     1150
1051    ACAGAAAACGCCAACCTCTGCGGTTGTTACGAACGTTTCCTAGTTACCACCAACTCCGAAAACGTCCTTTTCTATGTGAATTCCAGGGAATGCTCAGCTTTCCGT
1156    CAGGAGGGTTTAAACGAGCTTCTATTCGATTTGTGGCGGTTTCGACCCGATCAAGAATTATGTTCTACAGCACTTTCTATGCCATGAGGAGCGATGATTTCTCGT
1156      1160     1170     1180     1190     1200     1210     1220     1230     1240     1250
1156    GTCCTCCCAAATTTGCTCGAAGATAAGCTAAACACCGCCAAAGCTGGGCTAGTTCTTAATACAAGATGTCGTGAAAGATACGGTACTCCTCGCTACTAAAGAGCA
1261    CATTGTGTGGGCCTGTGATCGATGATGTCAAGCTTATAAGCGTTCGTAAACCATAGATGAATCACCAATCCTATTACAAGAAAATGCATCGTGTGGTCTTCTTTT
1261      1270     1280     1290     1300     1310     1320     1330     1340     1350     1360
1261    GTAACACACCCGGACACTAGCTACTACAGTTCGAATATTCGCAAGCATTTGGTATCTACTTAGTGGTTAGGATAATGTTCTTTTACGTAGCACACCAGAAGAAAA
1366    ATTTTCCTTAAACTGTCTTTGGTGTGTGTTGGATTTTATTGAAACGAGAAGGTGAAAAAAACAGAGTTTTATTTACGAGAGTTAGGGTTTTATATAGGCAGATGG
1366      1370     1380     1390     1400     1410     1420     1430     1440     1450     1460
1366    TAAAAGGAATTTGACAGAAACCACACACAACCTAAAATAACTTTGCTCTTCCACTTTTTTTGTCTCAAAATAAATGCTCTCAATCCCAAAATATATCCGTCTACC
1471    GCCTTTGCTTATATTGTATTTTCTTGTATGAGAAGGCAAAAGAAACGTTGCCTATATCTTATGTATCTAGGCCTTTTAATTTTTATGGAAGAGTTACAAAACTTA
1471      1480     1490     1500     1510     1520     1530     1540     1550     1560     1570
1471   CGGAAACGAATATAACATAAAAGAACATACTCTTCCGTTTTCTTTGCAACGGATATAGAATACATAGATCCGGAAAATTAAAAATACCTTCTCAATGTTTTGAAT
1576 CAATAAAGTTGTATGCATATTTTA
1576      1580     1590
1576 GTTATTTCAACATACGTATAAAAT
```

Primer:	Sequence:
BDXForward	5'-CTCTCGCTCACTCTTCTCCAA-3'
BDXReverse	5'-CGACAAAGCCTGAGAGTTCCC-3'
